# Perspectives on improving photosynthesis to increase crop yield

**DOI:** 10.1093/plcell/koae132

**Published:** 2024-05-03

**Authors:** Roberta Croce, Elizabete Carmo-Silva, Young B Cho, Maria Ermakova, Jeremy Harbinson, Tracy Lawson, Alistair J McCormick, Krishna K Niyogi, Donald R Ort, Dhruv Patel-Tupper, Paolo Pesaresi, Christine Raines, Andreas P M Weber, Xin-Guang Zhu

**Affiliations:** Department of Physics and Astronomy, Faculty of Science, Vrije Universiteit Amsterdam, Amsterdam 1081 HV, theNetherlands; Lancaster Environment Centre, Lancaster University, Lancaster LA1 3SX, UK; Carl R. Woese Institute for Genomic Biology, Department of Plant Biology, University of Illinois, Urbana, IL 61801, USA; School of Biological Sciences, Faculty of Science, Monash University, Melbourne, VIC 3800, Australia; Laboratory of Biophysics, Wageningen University, 6708 WE Wageningen, the Netherlands; School of Life Sciences, University of Essex, Colchester, Essex CO4 3SQ, UK; School of Biological Sciences, Institute of Molecular Plant Sciences, University of Edinburgh, Edinburgh EH9 3BF, UK; Centre for Engineering Biology, School of Biological Sciences, University of Edinburgh, Edinburgh EH9 3BF, UK; Department of Plant and Microbial Biology, University of California, Berkeley, CA 94720, USA; Howard Hughes Medical Institute, University of California, Berkeley, CA 94720, USA; Innovative Genomics Institute, University of California, Berkeley, CA 94720, USA; Molecular Biophysics and Integrated Bioimaging Division, Lawrence Berkeley National Laboratory, Berkeley, CA 94720, USA; Carl R. Woese Institute for Genomic Biology, Department of Plant Biology, University of Illinois, Urbana, IL 61801, USA; Department of Plant and Microbial Biology, University of California, Berkeley, CA 94720, USA; Howard Hughes Medical Institute, University of California, Berkeley, CA 94720, USA; Department of Biosciences, University of Milan, 20133 Milan, Italy; School of Life Sciences, University of Essex, Colchester, Essex CO4 3SQ, UK; Institute of Plant Biochemistry, Cluster of Excellence on Plant Science (CEPLAS), Heinrich Heine University, Düsseldorf 40225, Germany; Key Laboratory of Carbon Capture, Center of Excellence for Molecular Plant Sciences, Chinese Academy of Sciences, Shanghai 200032, China

## Abstract

Improving photosynthesis, the fundamental process by which plants convert light energy into chemical energy, is a key area of research with great potential for enhancing sustainable agricultural productivity and addressing global food security challenges. This perspective delves into the latest advancements and approaches aimed at optimizing photosynthetic efficiency. Our discussion encompasses the entire process, beginning with light harvesting and its regulation and progressing through the bottleneck of electron transfer. We then delve into the carbon reactions of photosynthesis, focusing on strategies targeting the enzymes of the Calvin–Benson–Bassham (CBB) cycle. Additionally, we explore methods to increase carbon dioxide (CO_2_) concentration near the Rubisco, the enzyme responsible for the first step of CBB cycle, drawing inspiration from various photosynthetic organisms, and conclude this section by examining ways to enhance CO_2_ delivery into leaves. Moving beyond individual processes, we discuss two approaches to identifying key targets for photosynthesis improvement: systems modeling and the study of natural variation. Finally, we revisit some of the strategies mentioned above to provide a holistic view of the improvements, analyzing their impact on nitrogen use efficiency and on canopy photosynthesis.

## Introduction

### By Roberta Croce

While photosynthesis is fundamental to life on Earth, powering the growth of plants and sustaining food chains, its solar energy conversion efficiency is surprisingly low, typically below 1% in crops. This low efficiency represents a significant opportunity for improvement, which is especially relevant in the context of a growing global population and pressure on available arable land to climate changes, with the consequent increase in agricultural demand. While substantial progress has been made in enhancing other aspects of crop yield, photosynthesis itself remains a largely untapped area for improvement. This is primarily due to the complexity of the photosynthetic process, which involves a multitude of genes and biochemical pathways. Unlike traits such as disease resistance or plant height, which can often be traced to a few key genetic changes and can be targeted by traditional breeding, the optimization of photosynthesis demands a deep molecular understanding and precise genetic manipulation. Only recently are the tools and knowledge for such interventions becoming available, making the direct improvement in photosynthesis a relatively new frontier in increasing crop productivity.

Can photosynthesis be improved? Recent proof-of-principle experiments have provided compelling evidence that it can. These studies have explored various innovative approaches, from introducing new biochemical pathways into plant systems to genetic modifications aimed at enhancing light-harvesting or carbon fixation efficiency. For instance, experiments have successfully demonstrated the feasibility of engineering plants to utilize light or assimilate carbon more efficiently or to bypass photorespiratory pathways, thereby boosting overall photosynthetic efficiency (e.g. Simkin et al. 2015; Kromdijk et al. 2016; South et al. 2019). These ground-breaking experiments show the potential to enhance photosynthesis and open new avenues for increasing crop productivity.

Why, after billions of years of evolution, are there still so many opportunities to enhance photosynthesis? Beyond the common understanding that plants evolve mainly for reproduction and survival rather than maximizing photosynthesis and productivity, there are additional factors. Climate changes, particularly changes in atmospheric carbon dioxide (CO_2_) levels, have made the photosynthetic properties of current plants less suited to modern environments. Enhancing ribulose 1,5-bisphosphate (RuBP) regeneration has been shown to increase photosynthesis in C_3_ plants (Lefebvre et al. 2005; Rosenthal et al. 2011), suggesting that the process may not have been fully optimized in response to new environmental conditions. Moreover, inefficiencies in some photosynthetic enzymes and structures may be evolutionary legacies. For example, Rubisco evolved when oxygen (O_2_) was scarce, making differentiation between CO_2_ and O_2_ less critical. As O_2_ became prevalent, increasing the CO_2_ specificity of Rubisco meant reducing its catalytic rate, potentially trapping it in a state of low specificity and efficiency (Erb and Zarzycki 2018). These factors suggest that contemporary photosynthesis systems, shaped by environmental changes and evolutionary history, hold potential for optimization and efficiency gains.

This perspective explores a range of promising strategies for improving photosynthetic efficiency. The strategies described here are at various stages of development; some have already demonstrated proof of principle, while others are still in the conceptual phase, collectively representing the state of the art in this field.

## Broadening the spectrum of plants to the far-red

### By Roberta Croce

**Figure 1.**
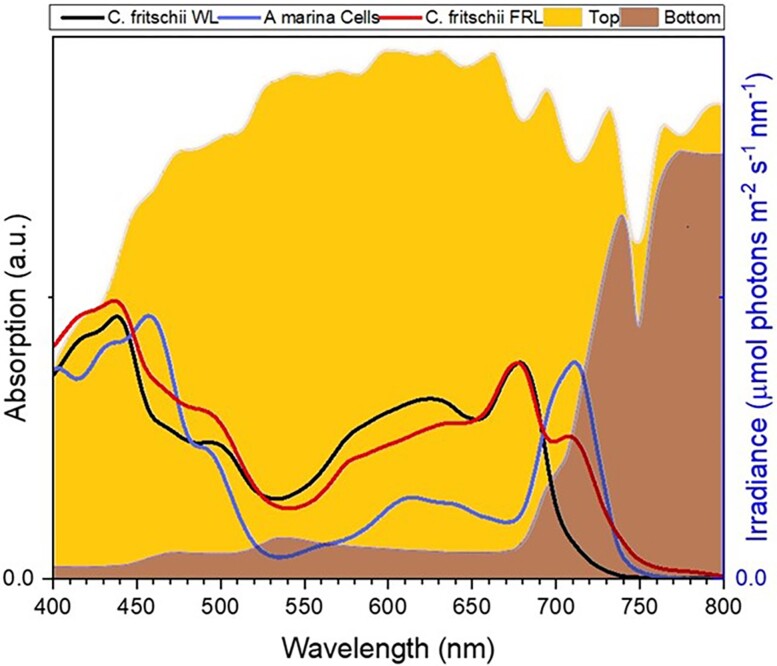
Solar spectrum on the top (yellow) and bottom (brown) of a crop canopy (adapted from Mirkovic et al. 2017). The absorption spectra of three cyanobacteria containing Chl *a* (black), Chl *a* and Chl *f* (red), and Chl *d* (blue) are also shown.

Light is the energy source of photosynthesis. However, only visible photons in the 400 to 700 nm range, the so-called range of photosynthetic active radiation, are used to power photosynthesis in most organisms, including plants. This limits the use of solar energy to <50% of what reaches the Earth's surface (Zhu et al. 2010). For a long time, it has been believed that photons above 700 nm would not carry enough energy to support efficient water oxidation. However, the discovery of cyanobacteria containing pigments absorbing in the far-red (FR) spectral region (Miyashita et al. 1996; Chen et al. 2010) has shown the feasibility of this process. It has thus been proposed that expanding the spectrum of plants up to 750 nm would lead to a gain of light absorption of around 20% (Blankenship and Chen 2013), which is a considerable increase in the energy available for growth. Making use of FR photons would be highly relevant for crops, as plants in the field are close together, and the light reaching the lower leaves is almost exclusively FR (Fig. 1) and currently cannot be used for photosynthesis, resulting in a close-to-zero photosynthetic rate at the bottom of a crop canopy (Srinivasan et al. 2017). The idea of broadening the absorption of crops to the FR is thus considered a promising strategy to improve their productivity (Slattery and Ort 2021; Wu et al. 2023).

How can this be achieved? Although this strategy has not yet been implemented in plants, its success in cyanobacteria serves as an excellent starting point for adapting it to crops. Three main factors are crucial for the efficient use of FR light in the first steps of photosynthesis: (i) the organism's capacity to harvest FR light, (ii) efficient transport of this energy to the photochemical reaction center (RC), and (iii) utilization of this energy for charge separation and stabilization of the charge-separated state. See Elias et al. (2024b) for a recent review.

Plants and algae use chlorophyll *a* (Chl *a*) and Chl *b* and carotenoids to harvest light. These pigments strongly absorb blue and red light but do not absorb in the FR. Some cyanobacteria, however, can synthesize two additional types of chlorophyll, called *d* and *f*, which absorb in the FR region of the spectrum (Gan et al. 2014; Ho et al. 2017). Both these chlorophylls differ from Chl *a* due to the presence of a formyl group in one of the substituents of the tetrapyrrole ring. This also means that a single enzyme is sufficient to transform the red-absorbing Chl *a* into the FR absorbing Chl *d* or *f*. While the enzyme for Chl *d* synthesis has not yet been discovered, the one for Chl *f* is known (Ho et al. 2016) and has been successfully heterologously expressed in cyanobacteria strains incapable of FR acclimation, showing that it leads to the production of Chl *f* (Shen et al. 2019b). These pigments then need to be coordinated to the photosynthetic proteins to form functional pigment–protein complexes. In FR light, alongside the Chl *f* synthase, cyanobacteria express paralogs of some/most photosynthetic proteins involved in pigment binding (Gan et al. 2014). These novel proteins have a higher affinity for Chl *f* in specific sites (Gisriel et al. 2021). However, it has already been shown that even the canonical photosynthetic proteins, such as the components of photosystem I (PSI), can bind Chl *f* (Tros et al. 2020) and that the light-harvesting complexes (LHCs) of plants, which are totally different from those of cyanobacteria, can accommodate both Chl *d* (Elias et al. 2021) and Chl *f* (Elias et al. 2024a).

In addition to the use of Chl *d* and *f*, FR light can also be absorbed by red-shifted Chls *a*. These are present in a small amount in PSI, also of plants, where they are called “red-forms”(Croce and van Amerongen 2013). The FR absorption spectrum of these pigments is due to the strong coupling between two or more Chls *a* (see Slama et al. 2023 for details) which is tuned by the proteins that determine the organization of the pigments. Designing FR Chl *a* binding sites can also be a strategy to enhance the FR absorption of plants without the need for additional Chl types.

After light is absorbed, the energy needs to be efficiently delivered to the RC for charge separation (see Croce and van Amerongen 2020 for more details). This process is made possible by the presence of proteins that organize the pigments. The plant photosynthetic units, PSI and photosystem II (PSII), are modular assemblies where a set of pigment–protein complexes act as an antenna responsible for harvesting light and transferring the excitation energy to the RC. The efficiency of this process hinges on the rate of energy transfer, as faster transfer reduces energy losses. The transfer rate depends on the inter-pigment distance, their mutual orientation, the magnitude of their excited-state transition dipole moments, and their excited-state energy landscapes. With Chl *d* and *f* having larger dipole moments than Chl *a*, and with the organization of the pigments within the complexes remaining largely the same in the studied cyanobacteria complexes (Gisriel et al. 2020, 2023; Hamaguchi et al. 2021), the key factor is the excited-state landscape. The substitution of all Chls *a* with FR absorbing Chls, as in the case of the cyanobacterium *Acaryochloris marina*, does not affect the rate of excitation energy transfer. On the contrary, integrating a few FR chlorophylls into complexes mainly containing Chl *a* can create local energy traps, slowing down the excitation energy transfer rate and reducing efficiency, as observed in cyanobacteria (Mascoli et al. 2020). Careful planning of the number and position of low-energy pigments is therefore crucial to ensure efficient delivery of the absorbed energy to the RC (Mascoli et al. 2022). This would also require the design of modified complexes where the affinity of Chl *d* and *f* is enhanced in specific binding sites.

The presence of low-energy chlorophylls in the RC can influence charge separation efficiency, as these Chls need to perform the same function as Chl *a*, but with less excited-state energy (Nürnberg et al. 2018). This can affect the recombination reactions and, in principle, even increase the possibility of photodamage. The regulation of the midpoint potential of the cofactors in the electron transport chain might be an issue (Viola et al. 2022). This aspect needs to be carefully studied in cyanobacteria to understand how they have overcome possible problems. However, while these factors might be critical for PSII, experimental results indicate that Chl *f*-containing PSI still works very efficiently with Chl *a* in the RC (Tros et al. 2021), suggesting that shifting PSI absorption to the FR might be easier than in the case of PSII.

Finally, the idea is to implement FR absorption in crops using the smart canopy concept, e.g. gradually increasing the absorption of FR photons in the lower part of the canopy (Ort et al. 2015). This allows for better use of light, as the upper leaves in a canopy, typically exposed to full sunlight, would have no advantage in absorbing FR light. In fact, having low-energy Chls in upper canopy leaves could decrease the overall efficiency of charge separation. On the contrary, the absence of FR pigments in the upper leaves allows the penetration of FR light to the bottom of the canopy, where leaves are light-limited and where it will be absorbed and used for photosynthesis. To create this absorption gradient across the canopy, we can again take inspiration from cyanobacteria. More and more species are being discovered that can undergo FR acclimation, meaning that these strains have a normal, Chl *a*-based, photosynthesis apparatus when exposed to white light and only express the Chl *f* synthase and paralogues of the photosynthetic proteins with enhanced ability to bind Chl *f* upon exposure to FR light (Gan et al. 2014; Gisriel et al. 2022). This process is controlled by phytochrome, a photoreceptor that senses the red/FR ratio and triggers the expression of FR-related subunits when needed (Ho et al. 2017). The same control should then be implemented in plants.

In summary, while the utilization of FR light in plant photosynthesis remains to be achieved, the expanding understanding of FR photosynthesis in cyanobacteria is paving the way for this application.

## Enhancing the efficacy of photosynthesis under field conditions: The case for antenna size reduction in crop canopies

### By Paolo Pesaresi

In C_3_ plant canopies, the photosynthetic machinery saturates at ∼25% of maximum solar flux, and this is one of the main factors that constrain productivity in these species (Jansson et al. 2010). Plants probably overinvest in light capture and synthesize high levels of thylakoid antenna proteins and associated chlorophylls because this increases their own fitness by depriving neighboring plants of light and nutrients (Freschet et al. 2011). However, since this trait is found in elite crops, it constitutes a major drawback for monocultures, in which all plants are expected to have the same fitness and yield capacity in order to maximize productivity in cultivated fields (Slattery and Ort 2021).

Reduction of chlorophyll content in leaves could offer a means to mitigate competition for light in monocrop stands, i.e. fields where one crop species is grown at a time, since the level and quality of the light that reaches leaves in the lower canopy should be higher and more uniform, thus boosting overall photosynthetic performance and yields (Blankenship and Chen 2013; Cutolo et al. 2023). Indeed, this strategy has been validated in several studies on cyanobacteria and microalgae (Nakajima and Ueda 1997; Beckmann et al. 2009; Perrine et al. 2012), although the same approach in higher plants has produced discordant results. A decrease in antenna size in tobacco (*Nicotiana tabacum*), for instance, led to an increase of about 25% in above-ground biomass accumulation under high-density cultivation conditions (Kirst et al. 2017). Similarly, beneficial effects were observed in a rice genotype with pale green leaves cultivated under high-light conditions (Gu et al. 2017a). Conversely, the few field studies that have used chlorophyll-deficient soybean (*Glycine max*) mutants showed a marked decrease in leaf mass accumulation and grain yield (Slattery et al. 2017; Sakowska et al. 2018; Genesio et al. 2020). These results underline the complexity of this trait, which is influenced by several factors including the gene/pathway involved, the degree of leaf chlorophyll reduction, the architecture of the crop canopy, as well as the environmental and growth conditions (Cutolo et al. 2023).

**Figure 2.**
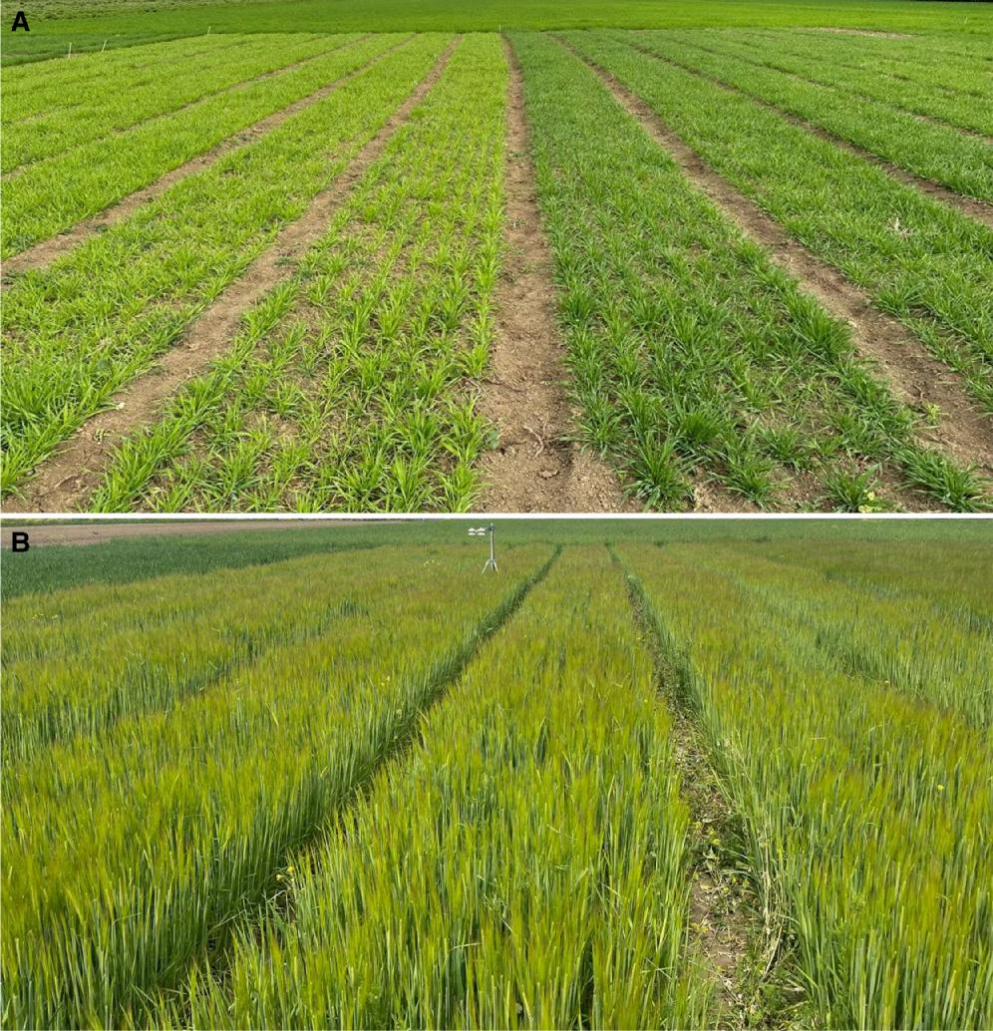
Phenotypes of the *hus1* mutant and control plants grown under field conditions at Azienda Agraria Sperimentale, Stuard (Parma, Italy). **A)** The *hus1* mutant and the control Sebastian variety at the tillering stage. **B)***hus1* plants at the heading stage. Courtesy of Lorenzo Genesio (National Research Council, Rome, Italy).

Recently, we have used the chemically induced mutant *happy under the sun 1* (*hus1*) to assess the impact of leaf chlorophyll reduction on biomass accumulation and grain yield in barley (Rotasperti et al. 2022). The pale green phenotype of *hus1* is due to a 50% reduction in the chlorophyll content of leaves, which enhances the efficiency of the conversion of light energy into chemical energy without increasing its susceptibility to photoinhibition (Fig. 2). The mutation introduces a premature stop codon in *HvcpSRP43*, which encodes the 43-kDa chloroplast signal recognition particle (cpSRP43) responsible for the delivery of antenna proteins to the thylakoid membranes (Klimyuk et al. 1999), and its truncation resulted in a decrease in the size of photosystem antenna complexes. Furthermore, the HvcpSRP43 protein has been shown to efficiently chaperone and stabilize glutamyl-tRNA reductase, a rate-limiting enzyme in tetrapyrrole biosynthesis, which enables the insertion of LHCs into thylakoids to be coordinated with chlorophyll biosynthesis (Wang et al. 2018). The dual role of HvcpSRP43 in chloroplasts makes *hus1* a rather specific mutant, since the reduced antenna size is coupled with a marked reduction in the activity of the tetrapyrrole biosynthetic pathway, which in turn suppresses the formation of highly toxic tetrapyrrole intermediates and pleiotropic photo-oxidative damage. Intriguingly, the *hus1* mutant accumulated biomass and grains at levels comparable to those observed for the control cultivar Sebastian, when grown under field conditions at standard density. These findings demonstrate that, when the selective pressure imposed by competition for resources in cultivated fields is attenuated, crops can indeed decrease their investment in antenna proteins and chlorophyll biosynthesis significantly, without detrimental effects on productivity.

Although *cpsrp43* knockout mutants showing the characteristic pale green phenotype have also been reported in other species, including rice (Wang et al. 2016), the *hus1* mutant was chosen for our investigations because a large set of functional genomics tools are available in barley (Rotasperti et al. 2020), making it an optimal model crop in which to test the performance of the several strategies reported to improve photosynthesis efficiency under realistic field conditions (Leister 2023; Smith et al. 2023). For instance, the Fast Identification of Nucleotide variants by droplet DigITal PCR technology developed by the Carlsberg Research Laboratory (Knudsen et al. 2022) provides a novel strategy for the rapid identification and isolation of targeted genetic variants in the elite spring barley cultivar RGT Planet. Thanks to this method, mutations that confer advantageous phenotypes on old varieties with low commercial value that carry several other single-nucleotide polymorphisms (SNPs) introduced by chemical mutagenesis—as in the case of the *hus1* mutant isolated within the *Hor*TILLUS population (Szurman-Zubrzycka et al. 2018)—can be transferred to commercially competitive varieties without the need for multiple, time-consuming (several years) backcrosses. Such an approach will make it possible to translate the major gains in biomass and grain yield reported in the last two decades as a consequence of the manipulation of photosystem antenna size in model species and crops, with tests mostly conducted in greenhouses or in small-scale field trials, into yield increases on farms. To this end, collaboration with plant breeders, agronomists, and crop physiologists is needed to select the most appropriate yield-testing protocols, including plot designs that avoid edge effects (which can distort yield estimates), definition of growing plant densities, and standard parameters to define yields (Khaipho-Burch et al. 2023). Such careful field trials will also allow us to test other major advantages thought to be associated with the pale green phenotype. Independent studies have predicted, for instance, that reductions in chlorophyll content should increase the efficiency of nitrogen use (Song et al. 2017; Sakowska et al. 2018). Similarly, simulations in soybean predict savings of up to 9% of leaf nitrogen upon a 50% reduction in leaf chlorophyll content (Walker et al. 2018). Furthermore, the development of pale green crops and the consequent increase in the fraction of reflected light, i.e. increased albedo, have been shown to mitigate the effects of heat waves triggered by global climate change (Genesio et al. 2021) and improve the efficiency of water use by reducing canopy temperature (Drewry et al. 2014). This latter aspect is supported by the finding that certain Syrian barley landraces and a few accessions of wild barley (*Hordeum vulgare* spp. *spontaneum*) in Israel, which are adapted to stable and very dry environments, are characterized by pale green leaves (Tardy et al. 1998; Watanabe and Nakada 1999; Galkin et al. 2018).

Overall, the time has come to translate photosynthesis research into the field, using barley as a model crop that can also exploit the availability of large collections of natural genetic diversity (Rotasperti et al. 2020). Numerous publicly funded organizations, such as the “Genomes to Fields Initiative” and the Consultative Group on International Agricultural Research (CGIAR), are conducting field trials that could contribute to achieving this goal with the medium-term objective of transferring this knowledge into other cereals, including wheat, given the high degree of conservation of the photosynthetic machinery in higher plants.

## Accelerating nonphotochemical quenching kinetics to improve photosynthetic efficiency

### By Dhruv Patel-Tupper and Krishna K. Niyogi

Photosynthesis needs photoprotection. Excess light can increase the lifetime of the singlet excited state of chlorophyll (^1^Chl*), resulting in higher yields of the longer-lived (∼ms) triplet excited state of chlorophyll (^3^Chl*) and photoinhibitory singlet oxygen (^1^O_2_*) (Niyogi 1999). ^3^Chl* and ^1^O_2_* can be quenched by carotenoids, but a ubiquitous first line of defense is the photosystem-scale de-excitation of ^1^Chl* and dissipation of excess absorbed light energy as heat—a suite of mechanisms that are measured (and referred to) as nonphotochemical quenching (NPQ) of Chl fluorescence (Bassi and Dall’Osto 2021).

**Figure 3.**
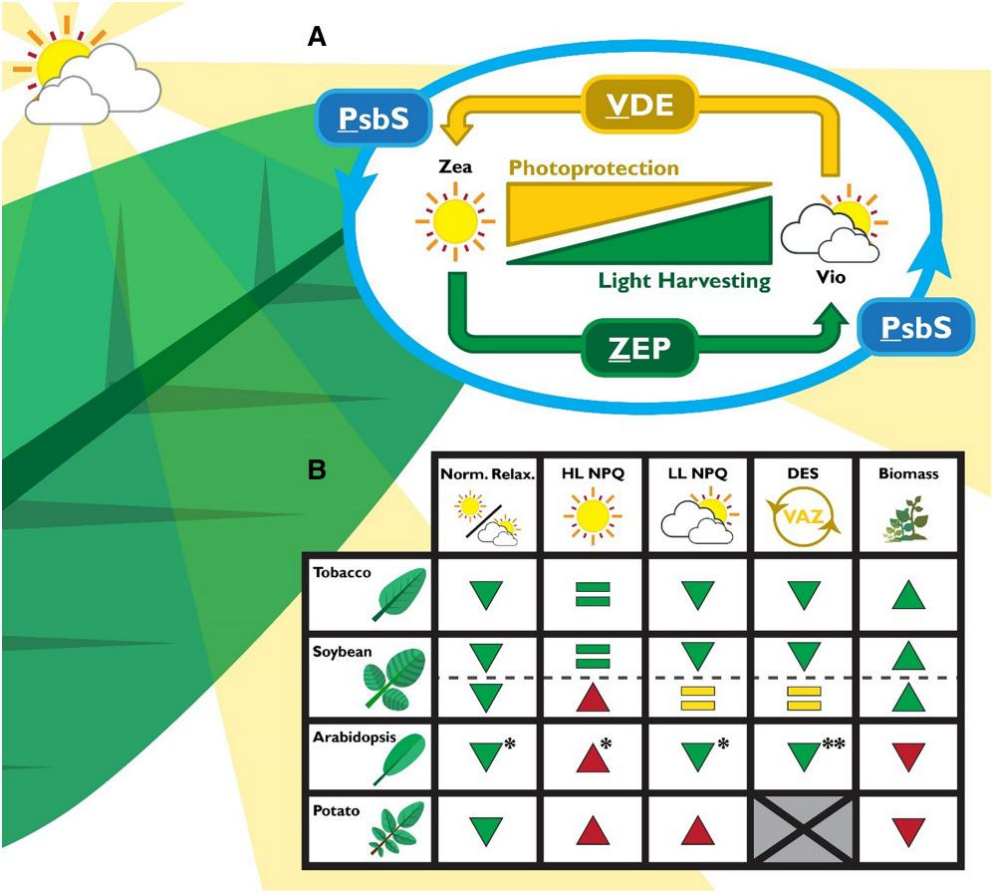
Engineering NPQ kinetics via VDE, PsbS, and ZEP overexpression. **A)** Schematic describing the relationships between VDE, PsbS, and ZEP in inducing and relaxing qE and qZ via the interconversion of zeaxanthin (Zea) and violaxanthin (Vio). **B)** Table summarizing differences in published VPZ phenotypes in tobacco (Kromdijk et al. 2016), soybean (De Souza et al. 2022), Arabidopsis (Garcia-Molina and Leister 2020), and potato (Lehretz et al. 2022), based on fluctuating light and field/growth chamber measurements. Triangles describe the directionality of the phenotype relative to wild type (WT). Green triangles indicate hypothesized beneficial photosynthetic efficiency phenotypes, maroon triangles indicate potentially deleterious phenotypes, and yellow lines describe neutral phenotypes. One asterisk (*) indicates NPQ phenotypes assessed at fluctuations of 500/50 *µ*mol photons m^−2^ s^−1^ rather than 2,000/200 *µ*mol photons m^−2^ s^−1^. Two asterisks (**) indicate phenotypes reported by Küster et al. (2023). Gray box indicates data not collected in its respective study.

NPQ comprises several components defined by their relaxation kinetics and molecular players. In plants, the rapidly induced, energy-dependent quenching (qE) requires the PSII subunit S (PsbS), the xanthophyll zeaxanthin (Zea), and a trans-thylakoid pH gradient (ΔpH), regulating quenching that is induced and relaxed on a seconds-to-minutes timescale (Bassi and Dall’Osto 2021). A slower type of Zea-dependent but ΔpH-independent quenching (qZ) operates on the timescale of several minutes (Nilkens et al. 2010). Zea is produced by violaxanthin de-epoxidase (VDE) and removed by zeaxanthin epoxidase (ZEP) in a xanthophyll cycle (Yamamoto et al. 1962) (Fig. 3A). The slowest components of NPQ include photoprotective qH (Malnoë et al. 2018) and photoinhibitory qI (Long et al. 1994), which relax on a timescale of hours. In concert, these highly conserved NPQ mechanisms provide the flexibility to cope with light fluctuations at varying intensities and timescales to sustain plant fitness.

Although NPQ protects the photosynthetic apparatus from excess light, it can also compete with and limit photosynthetic efficiency, especially in fluctuating light, where modeling has shown that slow relaxation of NPQ can limit CO_2_ assimilation by up to 30% (Zhu et al. 2004). Light intensity fluctuates by time of day, by local shading, and, most dramatically, by the movement of sunflecks in dense crop canopies (Slattery et al. 2018). Optimizing the light-dependent reactions of photosynthesis under dynamic, field-relevant light conditions is a rapidly expanding field with significant agronomic potential (Long et al. 2022; Leister 2023).

A transgenic approach to accelerate the relaxation of NPQ by overexpression of VDE, PsbS, and ZEP (hereafter VPZ, Fig. 3A) increased *N. tabacum* biomass by ∼15% (Kromdijk et al. 2016) and elite soybean seed yield by ∼20% (De Souza et al. 2022) in small-scale field trials. Although large-scale, multi-location field trials are still needed (Khaipho-Burch et al. 2023), these results point to NPQ as a novel potential target for crop improvement. However, the VPZ approach did not increase biomass in Arabidopsis (*Arabidopsis thaliana*; Garcia-Molina and Leister 2020) or yield in potato (*Solanum tuberosum*; Lehretz et al. 2022) under greenhouse and simulated fluctuating light conditions.

Here, we consider possible explanations for the varying VPZ results in different plants. Modeling suggests that the stoichiometries of the three proteins are critical (Zaks et al. 2012; De Souza et al. 2022), and thus, it may be necessary to optimize VPZ construct expression for each species depending on native qE and qZ capacities. Plant species with slower NPQ relaxation time constants (*τ*) and cultivars with higher yield potential may benefit the most from VPZ engineering efforts. Beyond species-specific factors, the literature to date suggests several key phenotypes that may be necessary to achieve greater photosynthetic efficiency by optimizing NPQ.

In all published VPZ studies (tobacco, soybean, Arabidopsis, and potato), transformants exhibited faster NPQ relaxation than wild type (WT), when normalized to high-light-acclimated NPQ. However, only in tobacco and two soybean lines (YZ-26-1C and ND-18-44) was faster relaxation also associated with the maintenance of WT high-light NPQ capacity. In other words, maximum NPQ in high light was also increased in VPZ soybean, Arabidopsis, and potato lines, which, except for soybean, was associated with decreased biomass relative to WT. The actual magnitude of residual NPQ in low light decreased only in tobacco, YZ-26-1C soybean, and Arabidopsis lines. Importantly, tobacco, soybean, and Arabidopsis lines showed increases in the low-light effective quantum yield of PSII (ΦPSII) (Fig. 3B).

While less residual NPQ and higher ΦPSII under fluctuating light are likely strong indicators of improved NPQ kinetics, it is apparent that these traits are not sufficient for increased biomass. Rigorous phenotyping that couple gas exchange measurements of CO_2_ assimilation with Chl fluorescence under field-relevant conditions is necessary to demonstrate the impact of faster NPQ kinetics, for example, using 2,000/200 *µ*mol photons m^−2^ s^−1^ fluctuating light assays that maximize fold changes in NPQ while replicating light fluctuations experienced in crop canopies (Slattery et al. 2018; Long et al. 2022). Measuring diurnal de-epoxidation states of the xanthophyll cycle (Kromdijk et al. 2016; De Souza et al. 2022) or the rate of re-epoxidation following HL-to-LL transitions (Küster et al. 2023) may be additional useful proxies for mitigation of the more slowly relaxing qZ component of NPQ. A recent analysis of ZEP-overexpressing lines of the stramenopile alga *Nannochloropsis* (Perin et al. 2023) indicates that for some species, there may also be room to reduce overall NPQ capacity while accelerating NPQ relaxation, highlighting the importance of species-specific phenotypic interrogation.

Natural variation in NPQ may provide nontransgenic avenues to breed for faster NPQ kinetics and higher photosynthetic efficiency. Genome-wide association studies for NPQ have previously revealed diversity in NPQ across rice subspecies and Arabidopsis ecotypes (Kasajima et al. 2011; Rungrat et al. 2019). Notably, both studies identified their strongest effect quantitative trait locus within the cis-regulatory regions upstream of the *PSBS* gene, suggesting NPQ as a trait that is subject to selection. Cowling et al. (2022) measured gas exchange and chlorophyll fluorescence phenotypes across 155 African rice accessions and found that shoot biomass was negatively correlated with NPQ induction capacity and positively correlated with NPQ relaxation rate, consistent with transgenic VPZ phenotypes associated with changes in biomass (Fig. 3B).

Several studies in soybean and maize (*Zea mays*) have identified potential loci for breeding for improved photosynthetic efficiency, using 41 nested association mapping soybean population parents (Wang et al. 2020), 751 diverse maize accessions (Sahay et al. 2023), and 320 multi-parent advanced generation inter-cross maize lines (Ferguson et al. 2023) across multiple years. However, in soybean, there appeared to be little quantitative variation in NPQ relaxation, with photosynthetic differences across lines primarily attributed to differences in Rubisco activation (Wang et al. 2020). Similarly, both maize studies did not resolve robust quantitative variation in residual low-light NPQ specifically, although differences across putative quantitative trait loci in NPQ capacity and maintenance of ΦPSII suggest that there may be diverse avenues for fine-tuning light-harvesting efficiency (Ferguson et al. 2023; Sahay et al. 2023). However, crop germplasm screens have not yet revealed differences in NPQ relaxation that are comparable to those in transgenic VPZ plants. Such results could suggest that there are tradeoffs associated with faster NPQ, or more simply, that faster NPQ relaxation has not been selected for in these crops.

Further efforts to improve NPQ kinetics should include accelerating recovery from the slowly reversible types of NPQ, qH, and qI. Nuclear expression of psbA, encoding the D1 subunit of PSII, to minimize qI associated with heat stress is one such approach with demonstrated success across several species (Chen et al. 2020). Additionally, we see tremendous potential in the use of gene editing of endogenous VPZ and other NPQ-related genes in crops to achieve desired NPQ phenotypes (Patel-Tupper et al. 2024). Fine-tuning of VPZ expression and testing diverse VPZ orthologues may be other avenues to bring NPQ-based improvements in photosynthesis closer to their theoretical potential.

## Increasing abundance of the cytochrome *b*_6_*f* complex to accelerate electron transport rate

### By Maria Ermakova

Chloroplast electron transport rate is a primary factor limiting photosynthesis. Classical steady-state quantitative models predict the rate of leaf photosynthesis over a range of intercellular CO_2_ partial pressures by considering which biochemical reactions are limiting in certain conditions. In the Farquhar–von Caemmerer–Berry model of C_3_ photosynthesis, carbon metabolism (CO_2_ availability, Rubisco function) limits assimilation at nonsaturating CO_2_ and electron transport limits assimilation under saturating light and saturating CO_2_ (intercellular CO_2_ partial pressure above 500 *µ*bar; Farquhar et al. 1980). Based on the model, at current atmospheric CO_2_ partial pressure (420 ppm, roughly corresponding to 200 *µ*bar intercellular CO_2_) C_3_ photosynthesis is primarily limited by the CO_2_ supply to Rubisco, but this limitation is anticipated to transition to electron transport when atmospheric CO_2_ exceeds 600 ppm. In the biochemical model of C_4_ photosynthesis, electron transport, Rubisco function, and regeneration of RuBP and phosphoenolpyruvate potentially co-limit assimilation under saturating light and saturating CO_2_ (intercellular CO_2_ partial pressure above 150 *µ*bar; von Caemmerer and Furbank 1999; von Caemmerer 2000). The vast difference in the saturating levels of CO_2_ between the two photosynthetic pathways is due to the metabolic C_4_ cycle of C_4_ photosynthesis acting as a carbon-concentrating mechanism. The C_4_ cycle operates across two cell types, mesophyll and bundle sheath cells (BSCs), and increases CO_2_ partial pressure in BSCs, where Rubisco resides, allowing Rubisco to work at maximum carboxylation rate. Crop models based on these metabolic models of photosynthesis predict yield increases for crops engineered with enhanced electron transport capacity up to 5.2% for wheat and 14.3% for maize (Harbinson and Yin 2023; Wu et al. 2023).

Early experiments on plants with genetically reduced electron transport components identified the thylakoid cytochrome (Cyt) *b*_6_*f* complex as the key regulator of the electron transport rate (Price et al. 1995, 1998; Yamori et al. 2011). The complex catalyzes plastoquinone oxidation, considered the slowest reaction of electron transport, and combines it with the translocation of protons across the thylakoid membrane, establishing the proton motive force for ATP generation (Tikhonov 2014). A low luminal pH makes proton translocation more difficult and therefore slows down Cyt *b*_6_*f* activity—a phenomenon known as photosynthetic control. Photosynthetic control via Cyt *b*_6_*f* plays an important role in photoprotection by limiting electron flow to PSI and thereby matching the output of the light reactions with the rates of ATP or NADPH consumption by carbon metabolism (Malone et al. 2021). Photosynthetic control therefore represents one molecular mechanism coordinating electron transport and carbon metabolism-related limitations of photosynthesis (Johnson and Berry 2021).

Since Cyt *b*_6_*f* was identified as a bottleneck of electron transport, efforts have focused on alleviating this limitation. In higher plants, the complex is comprised of seven essential subunits, of which Rieske FeS and PetM are nuclear-encoded (Schöttler et al. 2015). Enhancing the content of the Rieske FeS subunit through overexpression of the *petC* gene has been established as a method for increasing the abundance and activity of Cyt *b*_6_*f* in both C_3_ and C_4_ plants (Simkin et al. 2017b; Ermakova et al. 2019; Heyno et al. 2022). The increased abundance of the complex in transgenic plants has been confirmed through immunodetection of multiple subunits of the complex in leaves and of the whole complex in isolated thylakoids. Elevated Cyt *f* activity has also been detected in thylakoid membranes isolated from leaves of plants overexpressing Rieske FeS.

**Figure 4.**
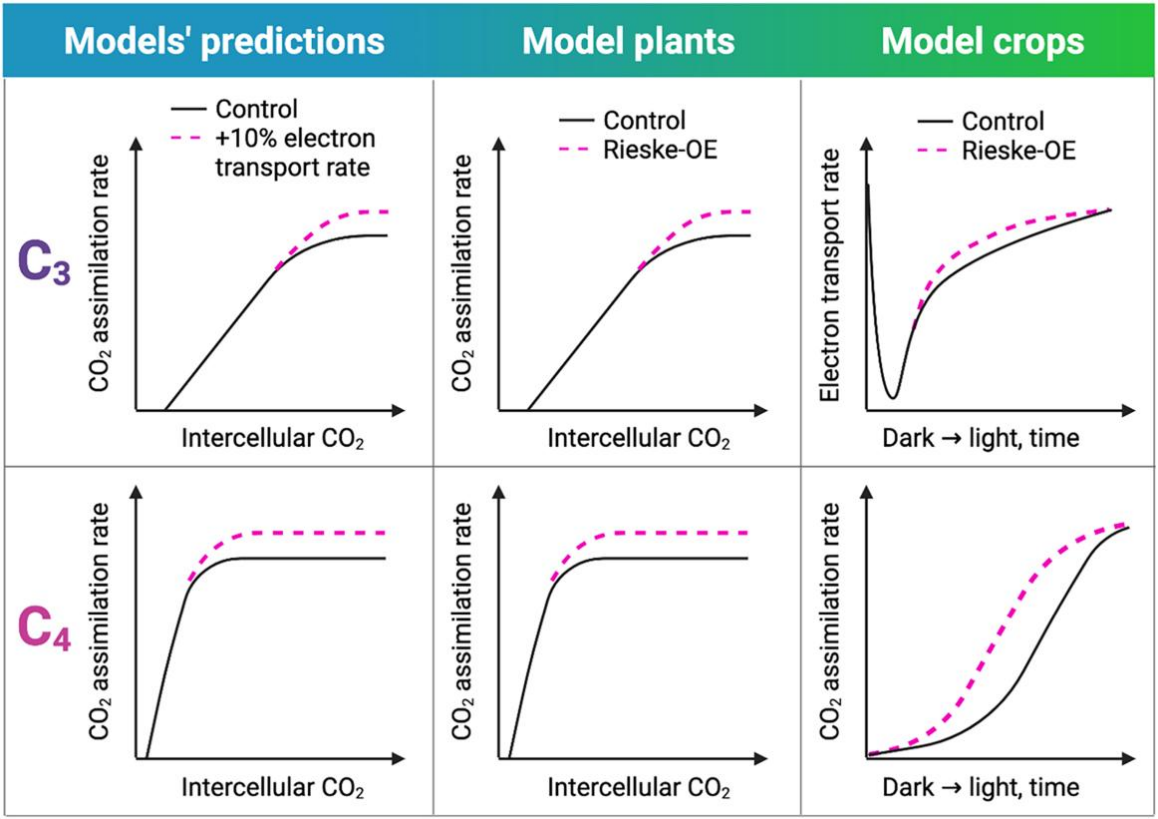
Increasing abundance of Cyt *b*_6_*f* to accelerate electron transport and enhance the rate of C_3_ and C_4_ photosynthesis: models’ predictions and results from plants overexpressing Rieske FeS subunit of Cyt *b*_6_*f* (Rieske-OE). Models’ predictions schematically depict simulations obtained with the C_3_ photosynthesis model of Farquhar et al. (1980) and C_4_ photosynthesis model of von Caemmerer and Furbank (1999). Schematic representations of Rieske-OE results are based on studies conducted in model C_3_ plant *A. thaliana* (Simkin et al. 2017b), model C_4_ plant *S. viridis* (Ermakova et al. 2019), model C_3_ crop *N. tabacum* Petit Havana (Heyno et al. 2022), and model C_4_ crop *S. bicolor* Tx430 (Ermakova et al. 2023). In model plants, in line with the models’ predictions, Rieske-OE stimulates steady-state electron transport, which results in increased CO_2_ assimilation rates at high light and nonlimiting CO_2_. In model crops, Rieske-OE provides transient increases of electron transport, which result in enhanced CO_2_ assimilation rate only in the C_4_ plant.

Increasing abundance of Cyt *b*_6_*f* in the model C_3_ and C_4_ plants, *A. thaliana* and *Setaria viridis*, results in faster electron transport rates through PSI and PSII. Furthermore, in line with the models’ predictions, a boost of electron transport capacity enables elevated CO_2_ assimilation rates in both model species (Fig. 4). These results confirm that Cyt *b*_6_*f* is a bottleneck of electron transport and that electron transport limits the rate of C_3_ and C_4_ photosynthesis at saturating light and saturating CO_2_. After genetically engineering plants with increased Cyt *b*_6_*f* content was identified as a promising route to improve photosynthesis, overexpression of Rieske FeS has been tested in model C_3_ and C_4_ crops, tobacco (*N. tabacum* cv. Petit Havana) and sorghum (*Sorghum bicolor* Tx430) (Fig. 4). No differences in steady-state CO_2_ assimilation rates and only transient increases of electron transport rate (seen as a faster re-oxidation of plastoquinone in the Q_A_ binding site of PSII upon increases of irradiance) are detected in tobacco with enhanced Cyt *b*_6_*f* abundance (Heyno et al. 2022). While in sorghum, increasing Cyt *b*_6_*f* content does not affect the steady-state rates of electron transport and CO_2_ assimilation, it does speed up the induction of photosynthesis, a process of light-induced activation of photosynthesis after a long period of darkness, and stimulates biomass and grain yield in glasshouse conditions (Ermakova et al. 2023). Overall, results of Rieske FeS overexpression in model crops suggest that the steady-state electron transport rate is no longer limited primarily by Cyt *b*_6_*f*.

The photosynthesis rate has undergone significant improvement during crop domestication and early breeding (Huang et al. 2022; Theeuwen et al. 2022). Therefore, it is conceivable that artificial selection of the biggest plants with the greatest yield may have unknowingly increased Rieske FeS content and improved the steady-state electron transport capacity in crops. However, overexpression of Rieske FeS in sorghum has still offered higher yield though transient increases of CO_2_ assimilation, highlighting the necessity of studying limitations of crop photosynthesis in dynamic light environments (Kaiser et al. 2017; Long et al. 2022). Looking forward, it is also essential to gain a better understanding of electron transport processes, especially in C_4_ plants, where electron transport chains of mesophyll and bundle sheath chloroplasts, distinct in their composition and outputs, cooperatively support seamless operation of biochemical pathways across the two cell types (Munekage 2016; Ermakova et al. 2021b). Cyt *b*_6_*f* is the first major bottleneck of electron transport to be successfully alleviated using genetic engineering, demonstrating the viability of this approach for crop improvement. Developing models with more detailed and mechanistic description of electron transport will be instrumental for identifying new targets for optimizing electron transport to boost productivity of crops (Johnson et al. 2021; Bellasio and Ermakova 2022).

## Improving the Calvin–Benson–Bassham cycle

### By Christine A. Raines

**Figure 5.**
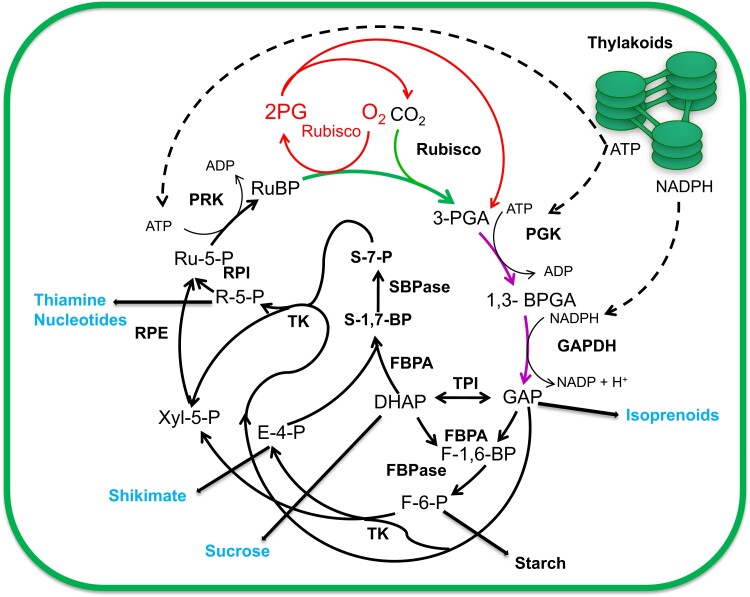
The CBB cycle. Energy in the form of ATP and NADPH (dashed lines) needed to drive the CBB cycle is produced in the thylakoid membrane located electron transport chain. The first step in the CBB cycle is carboxylation (green arrow) catalyzed by Rubisco resulting in the formation of 3-PGA. The next two reactions form the reductive phase (purple arrows) and are catalyzed by phosphoglycerate kinase, forming glycerate 1,3-bisphosphate (BPGA) using ATP and glyceraldehyde 3-phosphate dehydrogenase which forms glyceraldehyde-3-phosphate (GAP) consuming NADPH. Triose phosphate isomerase (TPI) catalyzes the production of dihydroxyacetone phosphate and together with GAP enters the regenerative phase of the cycle (black arrows) catalyzed by fructose 1,6-bisphosphate/sedoheptulose 1,7-bisphosphate aldolase (FBPA), forming sedoheptulose 1,7-bisphosphate (S1,7-BP) and fructose 1,6-bisphosphate (F1,6-BP). SBPase and fructose 1,6-bisphosphatase (FBPase) then produce sedoheptulose 7-phosphate (S7-P) and fructose 6-phosphate (F6-P) which are converted to 5C compounds in reactions catalyzed by TK, ribose 5-P isomerase (RPI), and ribulose 5-phosphate epimerase (RPE) resulting in the formation ribulose 5-P (Ru5P). The final step in the cycle is catalyzed by ribulose 5-phosphate kinase producing the CO_2_ acceptor molecule RuBP. The products of the CBB cycle are exported to several biosynthetic pathways for the biosynthesis of isoprenoids, starch, sucrose, shikimate, thiamine, and nucleotides. Rubisco has a competing oxygenase reaction, which results in the formation of 2-phosphoglycerate which enters the photorespiratory pathway (red arrows) (adapted from Raines 2003).

The Calvin–Benson–Bassham (CBB) cycle evolved over 2 billion years ago (Rasmussen et al. 2008) and is arguably the most important pathway on earth, capturing CO_2_ from the atmosphere and converting it into organic molecules that are used directly for the synthesis of isoprenoids, sucrose, starch, phenylpropanoids, thiamine, and nucleotides providing the basis for life on our planet. The CBB cycle is the primary biochemical pathway for the fixation of atmospheric CO_2_ in over 85% of plants, named C_3_ species, as the first stable product of this cycle is a three-carbon compound, glycerate 3-phosphate (Geiger and Servaites 1994; Sharkey 2019). In the 70 years since the CBB cycle was elucidated, it has been shown to be highly conserved across nature from cyanobacteria to the largest of land plants. The CBB cycle involves 11 enzymes and has three stages: carboxylation carried out by Rubisco, reduction, and RuBP regeneration (Fig. 5). Under light-saturating and CO_2_-limiting conditions, Rubisco activity is the major determinant of the efficiency of carbon fixation via the CBB cycle. However, as atmospheric CO_2_ levels rise and the light intensity decreases, this balance shifts, such that both the reductive and regenerative phases of the CBB cycle that catalyze the synthesis of the CO_2_ acceptor molecule RuBP become limiting. Improving photosynthesis has been identified as a target to increase crop yield based on theory, modeling, and empirical studies (Zhu et al. 2010; Simkin et al. 2019; Makino 2021; Raines 2022).

The complexity of the genetics and the biochemistry has made the Rubisco enzyme a challenging target for manipulation. Nevertheless, major efforts to improve photosynthesis have focused on improving Rubisco activity through both direct and indirect approaches. Direct approaches include protein engineering, directed evolution, natural variation screening, and manipulation of expression in transgenic plants (Parry et al. 2012; Yoon et al. 2020; Makino 2021; Gionfriddo et al. 2024). The introduction of CO_2_-concentrating mechanisms (CCMs) from algae and cyanobacteria and engineered synthetic pathways to bypass photosynthesis are indirect approaches being taken and are showing some promise (e.g. South et al. 2019; Shen et al. 2019a). This section will focus on reactions in the CBB cycle other than Rubisco; see the section below by Carmo-Silva for a discussion of Rubisco.

In the 1990s, antisense technology demonstrated that Rubisco did not have total control over CO_2_ assimilation under all conditions and identified sedoheptulose 1,7-bisphosphatase (SBPase), fructose 1,6-bisphosphate aldolase (FBPA), and transketolase (TK) as promising targets for the improvement in photosynthesis (Stitt and Schulze 1994; Raines 2003). Based on these studies, a transgenic overexpression approach has shown that increasing the levels of SBPase can improve photosynthesis and growth in algae and a number of plant species including: tobacco (in the field and greenhouse), wheat, and Arabidopsis; in contrast, no positive effect was observed in rice (Lefebvre et al. 2005; Simkin et al. 2015; Driever et al. 2017; Suzuki et al. 2019). Furthermore, tomato plants with increased SBPase activity were found to be more chilling tolerant with increased photosynthetic capacity (Ding et al. 2017). Overexpression of FBPA in tobacco also resulted in positive effects on photosynthesis and biomass (Uematsu et al. 2012; Simkin et al. 2015), and in tomato, an increase in seed weight in both optimal and suboptimal temperatures was observed (Cai et al. 2022). Introduction of the bifunctional cyanobacterial CBB cycle enzyme SBPase/FBPase into tobacco plants, lettuce, and soybean (in elevated CO_2_) has also resulted in improved CO_2_ assimilation and growth (Miyagawa et al. 2001; Tamoi et al. 2006; Kohler et al. 2017; Lopez-Calcagno et al. 2020).

Improvements in RuBP regeneration have also been realized through the introduction of additional proteins that function outside of the CBB cycle. Examples of this approach include combining expression of SBPase and FBPA with either ictB (a cyanobacterial membrane protein of unknown function previously shown to improve CO_2_ assimilation) or the H subunit of glycine decarboxylase (GDC) system (shown to increase CO_2_ fixation possibly through stimulating the photorespiratory cycle and reducing the negative impact of intermediates on the CBB cycle) in tobacco, which resulted in a further improvement in photosynthesis and growth over single-gene manipulations (Simkin et al. 2015, 2017a). Overexpression of either the endogenous SBPase enzyme or the bifunctional cyanobacterial SBPase/FBPase, together with the algal Cyt C6 protein, not only improved photosynthesis and yield but also increased water use efficiency when grown under field conditions (Lopez-Calcagno et al. 2020). A more recent example is the co-overexpression of SBPase with cytosolic FBPase in tobacco plants, resulting in improvements in biomass, plant height, stem diameter, and pod weight (Li et al. 2022), but in contrast co-overexpression of Rubisco with SBPase in rice did not result in an improvement in photosynthesis (Suzuki et al. 2019). Enhanced photosynthetic capacity was observed in plants in which the expression of a group of CBB cycle genes (FBA1, RCA1, FBP5, and PGK1) in response to higher levels of the Brassinazole resistant 1 transcription factor was increased (Yin et al. 2022). This result suggests that simultaneous overexpression of these enzymes may stimulate the CBB cycle.

Importantly as atmospheric CO_2_ rises, theoretical models predict that the limitation of carbon assimilation shifts from Rubisco to RuBP regeneration (Long et al. 2004). Therefore, modifications that improve RuBP regeneration are predicted to stimulate photosynthesis and yield under elevated atmospheric CO_2_. This is supported by experimental evidence using plants grown in free-air CO_2_ enrichment (FACE) facilities; when grown at 585 ppm CO_2_, transgenic tobacco plants overexpressing SBPase have greater yield increases at elevated CO_2_ (Rosenthal et al. 2011). When both CO_2_ and temperature are manipulated, transgenic overexpression of cyanobacterial bifunctional FBPA/SBPase in soybean protects against temperature-induced yield loss under elevated CO_2_ (Kohler et al. 2017). These results indicate that improving RuBP regeneration is one approach that could be used to mitigate the effects of climate change on yield, and also demonstrate the importance of testing manipulations in food crops under future climate conditions (Raines 2022).

Advances in kinetic flux and multi-scale modeling have provided novel predictions on how to further enhance the CBB cycle, and the application of rapid high-throughput and iterative approaches will be essential to identify the best candidates to achieve improvements to photosynthesis (Benes et al. 2020; Clapero et al. 2024). Synthetic biology may provide a route to build a completely synthetic, more efficient CO_2_ fixation pathway to operate in parallel with the endogenous cycle (Erb and Zarzycki 2016; Löwe and Kremling 2021) or to introduce improved enzymes to operate within the existing cycle. At the same time, new approaches enabling the identification of genetic factors and mechanisms involved in regulating the expression of CBB cycle genes will underpin the application of gene editing technologies to modify this pathway.

## Remarkable, integrated, and complex: paths to improving Rubisco in crops

### By Elizabete Carmo-Silva

Rubisco is imperfect yet unique and remarkable (Badger and Sharwood 2023). It catalyzes a rather complex set of reactions during the carboxylation of RuBP and can react with O_2_ rather than CO_2_, leading to oxygenation of the sugar phosphate substrate. It is the only carboxylase that uses substrates and products that are part of central plant metabolism and that is compatible with the CBB cycle (reviewed by Prywes et al. 2023b). Consequently, Rubisco is responsible for assimilating carbon from atmospheric CO_2_ into sugars, thereby enabling life on Earth. Rubisco's imperfections have been a research subject and a target for improvement since its discovery (Sharkey 2023). Because the enzyme carboxylates RuBP at relatively slow rates, plants invest large amounts of resources (especially nitrogen and carbon skeletons) into making Rubisco in sufficiently high abundance in the leaves of crop plants to support adequate rates of photosynthetic CO_2_ assimilation and plant growth (Carmo-Silva et al. 2015). The biogenesis of Rubisco, consisting of synthesis and assembly into the hexadecameric L_8_S_8_ form present in plants, is itself dependent on several ancillary proteins (Bracher et al. 2017), adding further demand on agricultural inputs and impacting resource use efficiency. Thus, while adequate abundance of Rubisco is a necessary consideration in strategies aimed at improving photosynthesis, it needs to be balanced with ensuring efficient use of resources to be compatible with sustainable agricultural practices.

Rubisco catalytic diversity investigations led to the suggestion that tradeoffs associated with the complex reaction mechanism limit the scope for Rubisco improvement (Tcherkez et al. 2006; Savir et al. 2010). A recent phylogenetic analysis demonstrated that, despite being among the 1% slowest evolving enzymes, the continual evolution of Rubisco was associated with improved catalytic efficiency and with greater leaf-level CO_2_ assimilation and photosynthetic nitrogen use efficiency (NUE) in C_3_ plants (Bouvier et al. 2024). Excitingly, a deep mutational scan using a Rubisco-dependent *Escherichia coli* strain revealed that several highly conserved residues of the loop 6, which folds over the catalytic site and is involved in substrate binding and catalysis, can be mutated without impacting catalysis, suggesting it is possible to target combinations of these residues for future Rubisco engineering efforts (Prywes et al. 2023a).

Rubisco catalytic sites are located at the interface between two large subunits (RbcL), and thus, RbcL has been considered the main source of catalytic diversity and the primary target for engineering efforts (Sharwood 2017). However, despite being far from the catalytic site, just a few changes in the Rubisco small subunit (RbcS) sequence can alter Rubisco catalytic properties (Lin et al. 2022). The role of RbcS in determining the abundance of Rubisco in leaves and adjusting catalytic efficiency has been recently reviewed by Mao et al. (2023) who highlighted the rise of RbcS as a target for improvement that is increasingly tractable as engineering approaches become more robust and widely applicable. Replacement of a crop plant Rubisco by a superior version such as that found in rhodophytes (Oh et al. 2023b) is exciting yet challenging given the need to express adequate levels of RbcS, RbcL, and compatible ancillary proteins. A more straightforward solution might be the use of fast-developing gene editing technologies to mutate specific residues required for enhanced catalysis, when we know what these are.

While the abundance and catalytic properties of Rubisco determine the maximum rate of carboxylation for a given leaf, the activity of Rubisco in crops is regulated by interaction with Rubisco activase (Rca), posttranslational modifications, and the chloroplast stroma environment, which can change rapidly in response to dynamic environmental conditions surrounding the leaf (Amaral et al. 2024). Under fluctuating light conditions, for example, photochemical, biochemical (including Rubisco regulation), and/or diffusional limitations can affect the efficiency of photosynthesis depending on the duration, frequency, and intensity of shade and full sun periods (Long et al. 2022).

Rca is a molecular chaperone that couples ATP hydrolysis with conformational remodeling of inhibited Rubisco catalytic sites, releasing sugar phosphate derivatives that occur naturally and bind tightly and unproductively to the enzyme (Bhat et al. 2017b; Mueller-Cajar 2017). Rca is itself regulated by the redox status, ATP, and Mg^2+^ availability in the chloroplast stroma, and some Rca isoforms activate Rubisco more efficiently under fluctuating light (Carmo-Silva and Salvucci 2013). The chaperone is thermolabile, and its ability to restore Rubisco activity is impacted at moderately high temperatures. Progress in understanding the mechanism of Rubisco activation by Rca (Hazra et al. 2015; Bhat et al. 2017a; Flecken et al. 2020) and identification of more thermostable and more efficient Rca isoforms suggest that improvement in Rubisco regulation to maximize carboxylation in current and future warmer climates is possible (Qu et al. 2023; Sparrow-Muñoz et al. 2023; Amaral et al. 2024). Significant unknowns exist in our understanding of Rubisco regulation by Rca, PTMs, and the chloroplast environment (Amaral et al. 2024) and the role of sugar phosphate derivatives and their phosphatases (Orr et al. 2023). Addressing these will aid identification of successful strategies for improving Rubisco, for sustainably increasing crop productivity and climate resilience.

**Figure 6.**
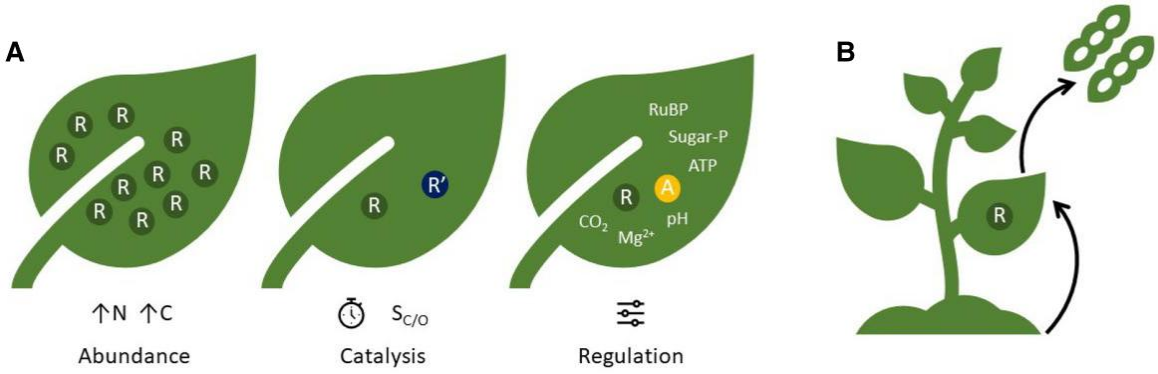
Improving Rubisco activity in crops. **A)** The activity of Rubisco in a crop leaf can be enhanced by increasing the enzyme abundance, improving its catalytic properties, or optimizing its regulation. Increasing the abundance of Rubisco requires more nitrogen and carbon allocation to Rubisco; some versions of Rubisco show faster rates of carboxylation or higher specificity for CO_2_ over O_2_; regulation of the enzyme activity can be enhanced by optimizing the interaction with Rca and ensuring the chloroplast stroma environment is favorable for carboxylation. **B)** To achieve Rubisco-driven crop yield improvements requires consideration of the whole plant. The canopy of the crop will determine which strategy is most promising to improve Rubisco and obtain increased yields and climate resilience. Coordination between photosynthetic sub-processes as well as a productive integration with central and specialized metabolism, plant development, and environmental responses is essential to ensure efficient and sustainable agricultural crop production in present and future climates. A, Rca; C, carbon; N, nitrogen; R, Rubisco; Sugar-P, sugar phosphate derivative.

Importantly, to maximize impact, any approach to improve photosynthesis should be considered holistically, and stacking of improvements in various reactions and sub-processes of photosynthesis will be required to ensure that other processes do not become limiting. For example, chloroplast electron transport is also sensitive to heat stress and has been shown to co-limit photosynthesis alongside Rubisco activation (Scafaro et al. 2023). Optimizing Rubisco activity in crop plants requires consideration of Rubisco abundance, Rubisco catalytic properties, and regulation of its activity (Fig. 6A), as well as how Rubisco activity interacts and is coordinated with other plant processes (Fig. 6B). The interaction with the regeneration of RuBP in the CBB cycle is most obvious, but abundance and properties of Rubisco should take into consideration integration with central and specialized metabolism, as well as the specific crop canopy architecture, the needs of the leaves at various canopy layers throughout crop development, and the remobilization of N and C skeletons stored in Rubisco into the crop product to be harvested.

## Introducing CCMs into plants

### By Alistair J. McCormick

The growth of many photosynthetic organisms, including most crops, is limited by the slow rate of CO_2_ assimilation by Rubisco and competition with O_2_ at the active site resulting in energetically wasteful photorespiration (Bauwe 2023). In response to the shortcomings of Rubisco, nearly every photosynthetic clade has evolved CCMs to supply Rubisco with concentrated CO_2_ and preferentially drive CO_2_ assimilation over photorespiration. CCMs can be broadly divided into two categories based on biophysical or biochemical processes. Biochemical CCMs initially capture CO_2_ as an organic metabolite before re-conversion to CO_2_ near Rubisco and include eukaryotic species that perform C_4_, C_2_, and crassulacean acid metabolism (CAM) photosynthesis (Lundgren 2020; Schiller and Bräutigam 2021; Furbank et al. 2023). Biophysical CCMs channel or actively pump in inorganic carbon (Ci, i.e. CO_2_ and HCO_3_^−^) to increase the intracellular Ci pool and include prokaryotic autotrophs, eukaryotic algae, hornworts, and seagrasses (Capó-Bauçà et al. 2022; He et al. 2023; Lafferty et al. 2023; Nguyen et al. 2023). All cyanobacteria and some chemoautotrophic bacteria sequester Rubisco within proteinaceous shells called carboxysomes, while almost all algae and some hornworts condense Rubisco into a micro-compartment called the pyrenoid. Both carboxysomes and pyrenoids are supplied with HCO_3_^−^ that is rapidly dehydrated by localized carbonic anhydrase (CA) activity to facilitate CO_2_ enrichment at the active sites of Rubisco.

**Figure 7.**
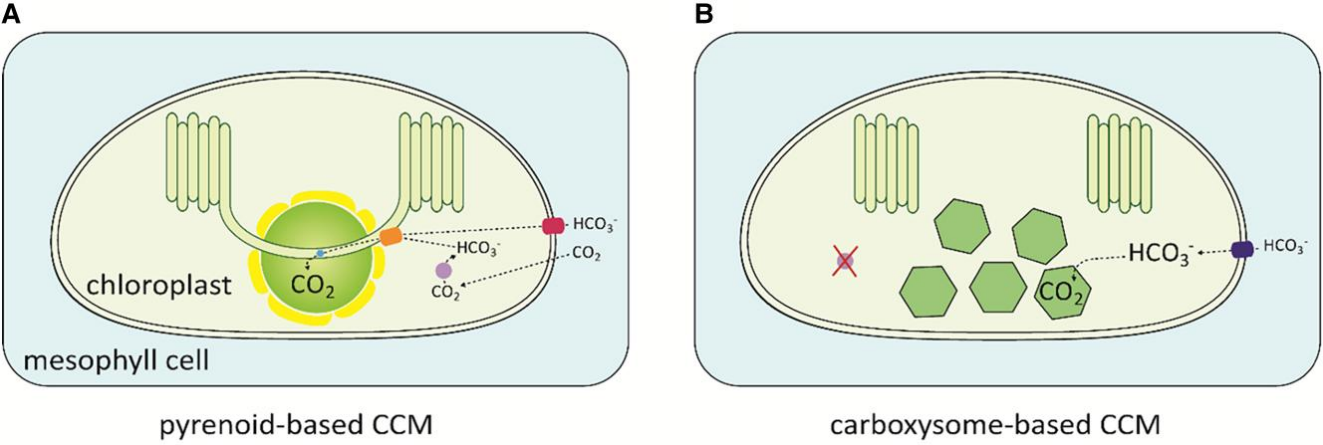
Overview of the requirements for introducing biophysical CCMs into plants. **A)** Engineering a functional pyrenoid-based CCM condensate into a MC chloroplast requires condensation of Rubisco with a linker protein, such as EPYC1 in Chlamydomonas, into a pyrenoid-like matrix (shown as a green sphere) that is traversed by thylakoid membranes containing a specialized lumenal carbonic anhydrase (CAH3, shown in blue) and bestrophin-like bicarbonate (HCO_3_^−^) channels on the thylakoid membrane (BST1-3, shown in orange). The addition of a diffusion barrier (e.g. a starch sheath shown in yellow) and an algal HCO_3_^−^ channel on the chloroplast envelope (LCIA, shown in red) are predicted to increase the efficiency of CO_2_ capture by Rubisco. **B)** A functional carboxysome-based CCM requires the correct assembly of carboxysome shells that encapsulate cyanobacterial Rubisco and a specialized carboxysomal CA (shown as green hexagons), and active bicarbonate (HCO_3_^−^) transporters (such as SbtA and/or BicA, shown in dark blue) on the chloroplast envelope that elevate stromal HCO_3_^−^ concentrations. Chloroplast stromal CA must be removed to prevent the loss of accumulated HCO_3_^−^.

The introduction of biochemical or biophysical CCMs into C_3_ crops is considered a high-risk high-gain engineering strategy to enhance crop yields and resilience (Fig. 7). Models have estimated that the theoretical gains in source leaf photosynthetic efficiency range from 30% to 60%, some of the largest improvements predicted for an engineering approach (Long et al. 2019). Although the levels of translation of such improvements into productivity are debated, for example, the importance of also considering sink demand to take full advantage of source enhancements (Paul 2021), a recent conservative model has predicted an 8% increase in wheat yields, specifically for successful introduction of the cyanobacterial CCM (Wu et al. 2023). Nevertheless, re-constituting any functional CCM in a C_3_ plant remains a complex endeavor, and engineering each CCM type is beset by specific challenges. In general, advances in our mechanistic understanding of different CCMs have proceeded in parallel with ongoing engineering efforts in a design build test learn (DBTL)-like cycle.

Research efforts to introduce biochemical CCMs into C_3_ plants have largely concentrated on engineering the C_4_ pathway. The C_4_ pathway is estimated to increase CO_2_ levels around Rubisco up to 10-fold above ambient (von Caemmerer and Furbank 2016), which provides C_4_ crops (e.g. maize and sugarcane) with increased photosynthetic efficiencies, growth rates, and in some cases higher water use efficiencies compared to C_3_ crops. Much of this work has been driven by the C_4_ rice project (Furbank et al. 2023), which has focused on introducing the specialized anatomical and biochemical traits of the model C_4_ crop maize into rice. Thus, C_4_ engineers have faced two complex challenges: construction of a “Kranz-like” C_4_ leaf anatomy with reduced vein spacing and increased organization between mesophyll and BSCs, and biochemical re-regulation for appropriate levels of cell-specific protein expression, for example, sequestration of Rubisco in the bundle sheath chloroplasts. Although the factors involved in the development of the Kranz anatomy are still not fully understood, advances in engineering biology have led to exciting progress in reconstituting C_4_ biochemistry in rice. This includes the generation and application of leaf cell-specific expression systems, such that several key C_4_ proteins can now be co-expressed and appropriately localized in rice from a single construct (Ermakova et al. 2021a). Attention is now also being expanded to the engineering potential of CAM and C_2_ pathways (Lundgren 2020; Schiller and Bräutigam 2021). The CAM pathway could facilitate increased drought tolerance, while C_2_ engineering offers some of the benefits of the C_4_ pathway with fewer anatomical modifications.

The most efficient biophysical CCMs are carboxysome-based CCMs (cCCMs) and pyrenoid-based CCMs (pCCMs), which can enhance CO_2_ concentrations around Rubisco by up to 1,000-fold and 40-fold, respectively (Price et al. 2013; Fei et al. 2022). Biophysical CCMs also function within a single cell, so transfer to C_3_ leaf mesophyll cells may be potentially simpler compared to introducing the C_4_ pathway. cCCMs have been consistently predicted to increase yield gains in C_3_ crops (Nguyen et al. 2023), in part because cyanobacteria have the fastest known form I Rubiscos, with carboxylation turnover rates (*k*_cat_) up to five times higher than C_3_ species (Ang et al. 2023). To date, good progress has been made in understanding the components involved in carboxysome assembly and in reconstructing α- and β-carboxysomes in plants (Borden and Savage 2022; Blikstad et al. 2023). Recently, Chen et al. (2023) reconstructed the α-carboxysome from the chemoautotroph *Halothiobacillus neapolitanus* in tobacco chloroplasts, which contained active heterologous Rubisco and CA enzymes. However, functional cCCMs in bacteria still require active uptake of HCO_3_^−^ and CA activity restricted to carboxysome (Price et al. 2013). Thus, testing whether carboxysomes can enhance growth in plants will require the removal of native chloroplastic CA activity and the introduction of functional HCO_3_^−^ transporters on the chloroplast envelope. Although the latter remains a long-standing challenge, new screening tools to test the functionality of active HCO_3_^−^ transporters and channels *in planta* could help to make progress in this area (Förster et al. 2023).

pCCMs are exclusively found in eukaryotes and are likely the most globally abundant CCM type. Pyrenoids are much larger than carboxysomes and characterized by highly diverse morphologies (Barrett et al. 2021), suggesting that there are many ways to achieve pyrenoid formation. The pCCM is best understood in the model alga *Chlamydomonas reinhardtii*, whose pyrenoid is characterized by three architectural features: a liquid-like phase-separated matrix of condensed Rubisco, pyrenoid tubules derived from thylakoid membranes that traverse the matrix to supply Ci, and a sheath of starch around the matrix that acts as a CO_2_ diffusion barrier (Ang et al. 2023; He et al. 2023). Modeling predicts that building a functional Chlamydomonas pCCM in a C_3_ plant chloroplast could increase CO_2_ assimilation rates by up to 3-fold (Fei et al. 2022). pCCMs do not offer the high carboxylation efficiencies of cCCMs but might be more readily plant-compatible based on the appropriate localization of pCCM components thus far expressed (Adler et al. 2022). Furthermore, pCCMs do not require the removal of chloroplastic CA activity and do not need active HCO_3_^−^ transport, at least at ambient CO_2_ levels (Fei et al. 2022). To date, Rubisco condensation into a “proto-pyrenoid” matrix has been achieved in Arabidopsis (Atkinson et al. 2020), with work ongoing to reconstitute the two remaining architectural features. Excitingly, recent work in diatoms and hornworts shows that a variety of solutions may exist to achieve a functional pCCM in plants (Lafferty et al. 2023; Nam et al. 2023; Oh et al. 2023a), for example, by employing a pyrenoid protein shell instead of a starch sheath. Future work could involve creative synthetic strategies that draw from aspects of all CCMs, such as prokaryotic HCO_3_^−^ transporters coupled to pyrenoids, or a biochemical C4 pathway localized within a single mesophyll cell (MC) where decarboxylation occurs in a modified chloroplastic carboxysome rather than the BS.

## C_3_-to-C_4_ transition and its potential for improving photosynthesis

### By Andreas P.M. Weber

**Figure 8.**
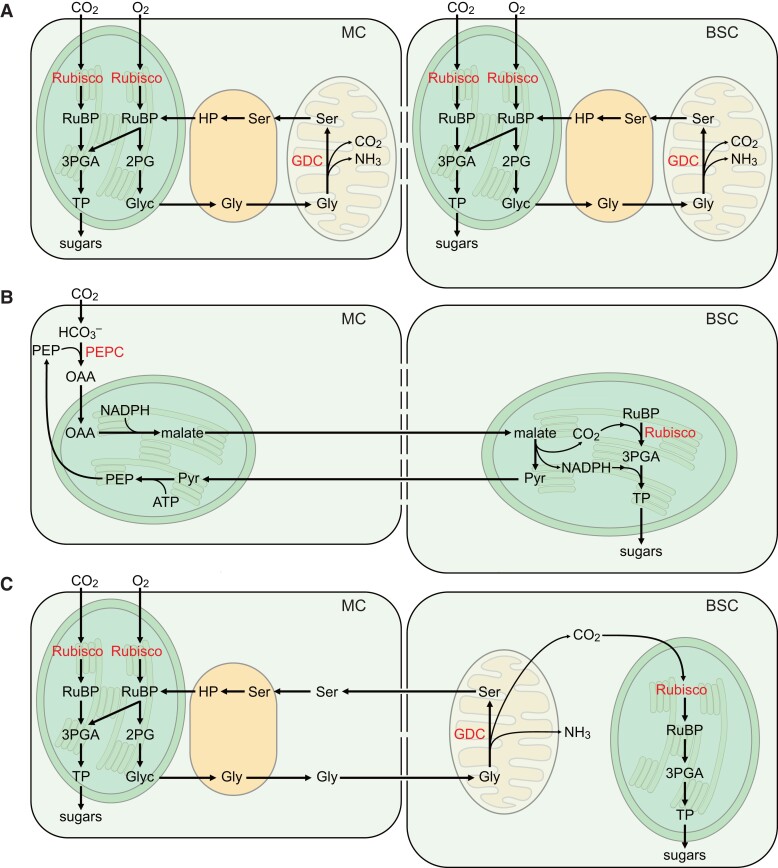
Schematic representation of C_3_, C_4_, and C_3_–C_4_ intermediate photosynthesis. **A)** C_3_ photosynthesis and photorespiration. Both mesophyll (M) and BSCs contain a fully functional photorespiratory pathway. **B)** C_4_ photosynthesis. The process of photosynthetic carbon assimilation is divided into two cell types, M and BSCs. M cells act as carbon pumps that increase the CO_2_ concentration in BSCs. In BSCs, the CBB cycle operates under elevated CO_2_ concentration, which reduces the rate of photorespiration [photorespiratory (PR) pathway not shown; the distribution of the PR pathway between cell types in C_4_ is likely equivalent to C_3_–C_4_ intermediate photosynthesis, as shown in Fig. 1C]. **C)** C_3_–C_4_ intermediate photosynthesis. Photorespiration is shared between M and BSCs, with mitochondrial glycine decarboxylation restricted to the BSCs. Glycine decarboxylation in BSCs locally increases the CO_2_ concentration and allows for a more efficient carbon assimilation in this cell type. Please note that many details, cofactors, and pathway intermediates are not shown for clarity. Clp, chloroplast; Gly, glycine; Glyc, glycolate; HP, hydroxypyruvate; Mito, mitochondrion Perox, Peroxisome; Pyr, pyruvate; Rubisco, ribulose 1,5-bisphosphate carboxylase/oxygenase; RuBP, ribulose 1,5-bisphosphate; Ser, serine; TP, triose phosphates.

In this section, we aim to provide a concise overview of photorespiration as a limitation on photosynthetic efficiency, the evolution of C_4_ photosynthesis via C_3_–C_4_ intermediates as an adaptation to low atmospheric CO_2_ conditions, and efforts to introduce C_4_ photosynthesis and bypasses of photorespiration into C_3_ plants as a means to increase photosynthetic efficiency. The efficiency of light energy conversion to biomass in C_3_ plant photosynthesis is limited by the rate of the oxygenation reaction of Rubisco. In the oxygenation reaction, the acceptor molecule of the CBB cycle, RuBP, is oxidized, not carboxylated, and one of the resulting metabolites, 2-phosphoglycolate (2PG), must be rapidly removed and recycled to 3-phosphoglycerate (3PGA) in a complex pathway called photorespiration (Bauwe 2023; Broncano et al. 2023). The recycling of two molecules of 2PG to 3PGA releases one molecule each of ammonium and CO_2_. It also consumes ATP and redox energy (Fig. 8). Walker et al. (2016) estimated that photorespiration reduces the yield of major C_3_ crops by 30% or more. Thus, suppression of photorespiration has great potential to increase crop yield (Eisenhut et al. 2019).

Over the past 20 My, more than 65 dicotyledonous and monocotyledonous plant lineages have independently and convergently evolved a more efficient form of photosynthesis called C_4_ photosynthesis (Sage et al. 2012). The frequent evolution of C_4_ is thought to have been triggered by a decline in atmospheric CO_2_ concentrations from >1,000 ppm to about 350 ppm (Sage et al. 2018). The decrease in CO_2_ was accompanied by a drying of the atmosphere and consequently increasing aridity in many parts of the world. Warm temperatures, reduced water availability, and lower atmospheric CO_2_ are conditions under which the oxygenation reaction of Rubisco is promoted and photosynthetic efficiency decreases.

C_4_ plants have a biochemical CO_2_ concentration mechanism that increases the CO_2_ concentration at the Rubisco site, thereby reducing the rate of photorespiration (Leegood 2002). The biochemical CO_2_ pump is supported by a characteristic leaf anatomy called the Kranz anatomy (Sedelnikova et al. 2018). Kranz anatomy is characterized by the formation of two concentric layers of photosynthetic cell types around the leaf vasculature. The inner ring, adjacent to the vasculature, consists of BSCs, which have a large cross-sectional area and are densely filled with chloroplasts containing Rubisco and operating the CBB cycle. BSCs usually perform predominantly cyclic photosynthetic electron transport and have low activity of PSII and hence linear electron transport. The outer ring consists of mesophyll cells, which are always associated with BSCs and face the leaf air space. Mesophyll cells typically contain fewer chloroplasts than BSCs, and they contain little or no Rubisco. MC chloroplasts are capable of linear photosynthetic electron transport. The vascular-bundle sheath-mesophyll (V-BSC-M) cell cluster is the basic unit of a C_4_ leaf and is repeated, resulting in a repeating V-BSC-M-M-BSC-V pattern throughout the leaf.

Mesophyll cells serve as the first carbon-fixing cells. CO_2_ from the leaf airspace enters the MC and is converted to HCO_3_^−^ by CA. HCO_3_^−^ reacts with phospho*enol*pyruvate, catalyzed by phospho*enol*pyruvate carboxylase (PEPC), to form the C_4_ acid oxaloacetate (OAA). OAA is further converted to malate and/or aspartate, which diffuses along their concentration gradient to the BSCs. In the BSCs, malate and/or aspartate is decarboxylated by one of three decarboxylating enzymes (or a combination thereof; Fig. 1B). These are mitochondrial NAD-malic enzyme (NAD-ME), chloroplastic NADP-malic enzyme (NADP-ME), and PEP carboxykinase (PEPCK). The predominant pathway of decarboxylation defines the C_4_ subtype (NAD-ME, NADP-ME, or PEPCK), although it is controversial whether PEPCK is a distinct subtype or a complementary pathway to either NAD-ME or NADP-ME (Wang et al. 2014a). The C_4_ carbon pump raises the CO_2_ concentration in BSCs to >1,000 ppm, which suppresses the oxygenation reaction of Rubisco to low levels. However, we emphasize that photorespiration is essential in C_4_ plants, as indicated by the lethal phenotype of mutants in the C_4_ photorespiratory pathway (Zelitch et al. 2009; Levey et al. 2019).

Based on physiological, anatomical, and biochemical data, as well as computational modeling, it has been proposed that C_4_ photosynthesis has gradually evolved from the ancestral C_3_ state through C_3_–C_4_ intermediate states (Sage et al. 2018; Schlüter and Weber 2020). The basic concept is that the photorespiratory pathway loses cell autonomy and is instead split between mesophyll and BSCs (Hylton et al. 1988; Sage et al. 2014). The CO_2_ (and ammonia) liberation step of photorespiration, catalyzed by GDC in the mitochondria, ceases to function in mesophyll cells and is restricted to BSCs (Hylton et al. 1988; Rawsthorne et al. 1988). Glycine decarboxylation in BSCs locally increases the CO_2_ concentration in this cell type, thereby increasing the efficiency of Rubisco in BSC chloroplasts.

It has been estimated that under conditions that promote high rates of photorespiration (high temperature, low leaf-internal CO_2_ concentration due to, e.g. low stomatal conductance), CO_2_ accumulates in BSCs at two to three times the level in the mesophyll (Bauwe et al. 1987; Keerberg et al. 2014). Glycine decarboxylation by GDC also releases ammonia, which must be refixed to avoid ammonia toxicity and returned to mesophyll cells to maintain nitrogen balance. It has been proposed that carbon skeletons for ammonia shuttling are provided by mesophyll cells in the form of malate, forming a low-level C_4_ metabolic cycle (Mallmann et al. 2014). In this scenario, malate is oxidized to OAA by NADP-malate dehydrogenase. OAA is subsequently transaminated to aspartate, and aspartate is then transported back to the mesophyll cells. Additionally, a portion of the malate can undergo decarboxylation, forming pyruvate, which can be transaminated into alanine, providing an alternative nitrogen transport mechanism to the mesophyll cells. Both malate oxidation via NADP-MDH and oxidative decarboxylation through NAD(P)-ME are facilitated by a predominantly oxidized NAD(P) pool (Bräutigam et al. 2018). If PSII activity is lost from BSCs, it would lead to an increase in the oxidized plastid NADP+ pool. This, in turn, would further promote the evolution toward a full C_4_ carbon fixation.

Many staple crops, such as rice and wheat, use C_3_ photosynthesis. Given the high efficiency of C_4_ photosynthesis, it has been proposed that conversion of these crops to C_4_ would result in large yield gains (Hibberd et al. 2008). Indeed, the analysis of a rice new plant type (Sheehy et al. 2001) with large panicles and a low number of tillers showed a grain formation efficiency of only 42%, i.e. a substantial number of juvenile spikelets were not converted into mature, filled spikelets. This finding indicated that rice yield is not limited by sink size, but rather by source strength, i.e. the capacity of leaves to produce and export photo-assimilates (Sheehy et al. 2001). Based on these data, it was estimated that rice yield could be doubled if source strength was increased appropriately. To enhance the source strength, the conversion of rice into a C_4_ plant was proposed as a strategic approach, with the potential to increase yield by 50%. This proposition has gained support through FACE experiments. When elite rice varieties were cultivated in FACE conditions with 200 ppm CO_2_ concentrations higher than the ambient levels, they produced 13% more grain yield (Zhang et al. 2013). This suggests that improving photosynthesis and thus source strength directly contributes to higher yields in rice.

Significant progress has been made in advancing C_4_ photosynthesis in rice (Ermakova et al. 2020). Activation of photosynthetic organelles in the rice BS was achieved by constitutive expression of the maize transcription factors GOLDEN2 or GOLDEN2-LIKE (Wang et al. 2017). Furthermore, five minimal C_4_ cycle genes (NADP-ME, PEPC, PPDK, MDH, and CA) were successfully expressed in rice from a single construct (Ermakova et al. 2021a). The respective gene products were found to be expressed in the correct cell types and subcellular compartments. [^13^C]–CO_2_ labeling showed that some of the labeled carbon is directed through PEPC to malate and aspartate. Rapid labeling of citrate indicated movement of the introduced label toward the tricarboxylic acid (TCA) cycle, while no label was detectable in CBB cycle intermediates. The absence of [^13^C] label in the CBB intermediates indicates that either labeled malate does not enter the plastid stroma or that there is little malate decarboxylation via plastidic NADP-ME (Ermakova et al. 2021a). These results suggest that expression of the core C_4_ metabolic enzymes alone is unlikely to be sufficient to establish a functional C_4_ cycle. Further development of C_4_ rice will likely require the co-expression of organellar metabolite transporters, some of which are unknown. Additionally, it is crucial to prevent the diversion of recently fixed carbon into the TCA cycle, and it may be necessary to inhibit photosynthetic linear electron transport in BSCs to achieve the oxidized NADP+ pool required for decarboxylation by NADP-ME (Bräutigam et al. 2018). Recently, a single promoter TALE system has been reported for tissue-specific expression of multiple transgenes in rice (Danila et al. 2022). In this system, a single cell-specific promoter drives the expression of a synthetic designer transcription activator-like effector that can bind to synthetic TALE-activated promoters (Brückner et al. 2015). This means that multiple genes can be expressed from a single cell-specific promoter in the desired cell type. This technological advance overcomes previous limitations associated with the limited choice of cell-specific promoters for transgene expression in rice and will facilitate transgene stacking, which is critical given the substantial number of transgenes required to implement the trait.

Efforts to establish C_4_ photosynthesis (or any other C_3_ crop) are ongoing and showing significant progress. In the meantime, other approaches to increase photosynthetic carbon gain in rice and other crops have been explored (Smith et al. 2023). The introduction of synthetic bypasses to photorespiration in rice has been associated with yield increases of up to 15%, which is close to what has been observed in FACE experiments (Shen et al. 2019a). Furthermore, it may be sufficient to install a basic C_3_–C_4_ intermediate photorespiratory carbon pump in rice, as metabolic modeling suggests that C_3_–C_4_ intermediate photosynthesis increases carbon gain over a wider range of environmental conditions than C_4_, which is most advantageous under high-light and high-temperature conditions (Bellasio and Farquhar 2019).

## Manipulating stomatal features to improve photosynthesis and water use efficiency

### By Tracy Lawson

Gaseous exchange between the leaf interior and the external atmosphere is determined by stomatal conductance (g_s_), and therefore, stomatal regulatory mechanisms play a pivotal role in determining the rates of photosynthetic carbon assimilation (*A*) and the loss of water through transpiration. Both of these physiological processes hold paramount significance for overall plant performance, productivity, and yield. The importance of CO_2_ uptake for photosynthesis is self-evident; however, water loss is equally vital for evaporative cooling and the maintenance of optimal leaf temperature to facilitate photosynthesis (Long et al. 2022), as well as transpiration driving uptake of essential nutrients from the soil to the aerial parts of the plant. The regulation of stomatal aperture, which balances CO_2_ uptake and water loss, is thus fundamental in determining plant water use efficiency, determined as A/E or intrinsic water use efficiency (W_i_) when assessed directly as a function of g_s_, (A/g_s_) (Lawson et al. 2010). The significant impact stomata have on photosynthetic processes has led to increasing recognition of their potential as valuable targets for manipulation, to enhance crop performance, as well as to develop future crops that can tolerate the challenges posed by climate change (Matthew and Lawson 2019).

**Figure 9.**
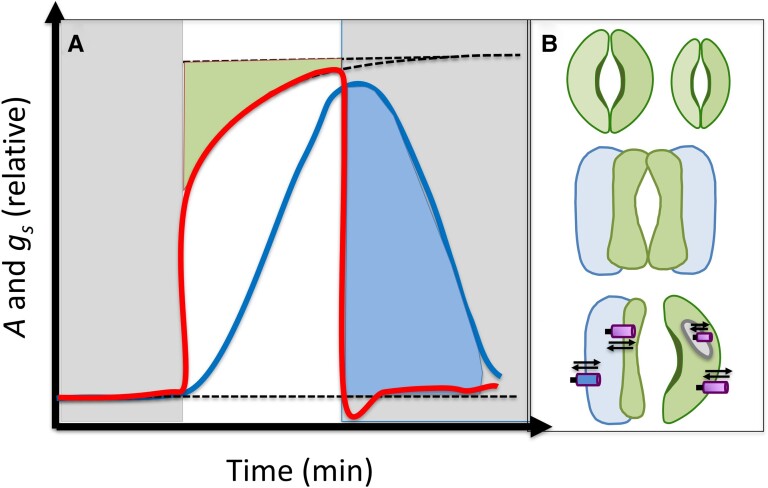
Schematic diagrams. **A)** Illustrating kinetic responses of A and g_s_ to a change in light intensity from low (gray shading) to high intensity (white area). Stomatal responses (blue line) are an order of magnitude slower than A (red line). The shaded green area represents lost CO_2_ due to diffusional constraints of slow stomatal opening, while the blue shading represents unnecessary water loss as a result of slow stomatal closure. **B)** Illustrating known mechanisms that increase the rapidity of g_s_ responses, including smaller stomata (top), dumbbell-shaped GC (middle), and manipulation of ion transport between GC and SC, at both the plasma membrane and the tonoplast (bottom).

Stomatal conductance is the product of both anatomical features and biochemical factors, providing several research avenues for exploitation and manipulation for improved performance. Manipulation of stomatal density (SD) through changes in expression of key genes in the stomatal development and/or patterning pathways has clearly illustrated their potential to either increase the rate of carbon assimilation through removing stomatal limitation (Tanaka et al. 2013; Buckley et al. 2020) or enhance the water use efficiency (W_i_; Bertolino et al. 2019). Although there are various approaches to manipulating stomatal numbers (Bertolino et al. 2019), genes within the epidermal patterning factor (EPF) and epidermal patterning factor-like (EPFL) family have been a particular focus (Harrison et al. 2020). Overexpression of EPF1 and 2 has demonstrated that a reduction in SD (and conductance) resulted in improved drought tolerance and increased water use efficiency (Bertolino et al. 2019; Leakey et al. 2019). However, decreased stomatal conductance usually lowers the rate of carbon assimilation; therefore, it is intriguing that reducing SD (by between 46% and 58%) in the key C_3_ crops, rice and wheat (Caine et al. 2019; Dunn et al. 2019) did not impose any diffusional constraints on carbon assimilation. Overexpressing EPFL9/STOMAGEN increases SD leading to a greater conductance associated with enhanced A (Tanaka et al. 2013; Sakoda et al. 2020), highlighting the impact of diffusional constraints on carbon assimilation (Lawson et al. 2012). However, it is important to note that while augmenting SD to increase stomatal conductance may appear promising for removing diffusional constraints (Sakoda et al. 2020), increased water loss erodes water use efficiency (Lawson and Blatt 2014). Furthermore, high SD has been linked to stomatal clustering and impaired stomatal kinetics, resulting in reduced stomatal conductance and rates of carbon assimilation (Dow et al. 2014). These effects are attributed to reductions in critical ion channels necessary for guard cell (GC) osmoregulation and pore opening, such as reduced K^+^ channel activity (Papanatsiou et al. 2016). Therefore, while the manipulation of SD presents a promising avenue for modifying stomata conductance, it is becoming evident that future endeavors should adopt a more holistic approach that considers both anatomical aspects and functional attributes such as stomatal kinetics (Fig. 9).

The close correlation between the rate of carbon assimilation and stomatal conductance (under steady-state conditions) is well established (Wong et al. 1979); however, it is not always constant (Lawson and Morison 2004), and under dynamic conditions, stomatal responses to changing environmental conditions are an order of magnitude slower than the rate of carbon assimilation, leading to a temporal disconnect between these two processes (Lawson and Blatt 2014). Slow stomatal opening has been reported to reduce carbon assimilation by ca. 10% across a range of species, while slow closure leads to unnecessary water loss that can erode water use efficiency by up to 50% (McAusland et al. 2016). Significant species and cultivar differences in the kinetic responses of stomata that depend on anatomy and biochemistry have been demonstrated (McAusland et al. 2016). This research has led to increasing interest in the rapidity of stomatal conductance and GC regulation as novel targets for improving carbon assimilation and water use efficiency (Lawson and Vialet-Chabrand 2019). Recently, Papanatsiou et al. (2019) used optogenetics to enhance solute fluxes in GCs, accelerating stomatal opening and closing, and verifying that enhancing stomatal kinetics improved water use efficiency with no carbon penalty. Engineering ion channels involved in K+ fluxes in the GCs has further demonstrated the potential to increase the kinetic responses of stomatal conductance and improve water use efficiency (Horaruang et al. 2022). GC metabolism also holds the promise of offering numerous innovative targets for manipulating both the rapidity of stomatal responses and the coordination between carbon assimilation and stomatal conductance (see review by Lemonnier and Lawson 2023).

Smaller stomata have been proposed to have faster kinetics (Drake et al. 2013), most likely facilitated by the greater surface area to volume ratio enabling more rapid solute and water exchange between GCs and the surrounding cells (Hetherington and Woodward 2003). However, to date little is known about the genetic regulation of stomatal size, and no key targets have been identified for manipulation. Stomatal size has been linked to stomata density, with smaller stomata associated with higher density (Drake et al. 2013); therefore, it may not be possible to manipulate size independently of density. The shape of the GCs and cells that surround them (called subsidiary cells) also influence the speeds at which stomata open and close (Franks and Farquhar 2007). Raissig et al. (2017) confirmed the importance of subsidiary cells in *Brachypodium distachyon* mutants lacking these cells, which resulted in slower kinetics and reduced stomatal conductance. These findings have opened up subsidiary cell metabolism and transport as a potential new avenue of research to explore novel targets for increasing stomata kinetics (Gray et al. 2020) (Fig. 9).

While the scope of this perspective may not encompass all recent advancements, it is worth noting that numerous exciting developments in stomatal research could provide new opportunities to improve crop performance in a changing climate. Nonfoliar stomatal behavior is gaining increasing attention due to the significant impact on photosynthesis and crop yield (especially during periods of plant stress). Additionally, current work has demonstrated considerable water loss associated with these nonfoliar tissues, providing possible new targets to improve whole-plant water use (Lawson and Milliken 2022). While most research primarily concentrates on leaf-level stomatal responses, recent studies have unveiled differences in stomatal kinetics between the adaxial and abaxial stomata (Wall et al. 2022). This discovery opens up the potential to independently manipulate these two surfaces, if the genes responsible for the stomatal patterning on each surface can be elucidated. However, if we are to exploit the potential targets highlighted in this perspective we need a greater understanding of GC signal transduction pathways, metabolism, and the mechanisms that coordinate carbon assimilation and stomatal conductance.

## Natural variation in intrinsic yield potential

### By Jeremy Harbinson

The model of Z-scheme oxygenic photosynthesis, with the CBB cycle fixing CO_2_, is the dominant photosynthetic process of the biosphere, and as such, it provides most of the energy and biomass that supports life on Earth. The consistent use of a Z-scheme CBB cycle photosynthetic engine means that there is little variation in the basic physiological and biochemical mechanisms of photosynthesis. However, since the colonization of land by the embryophytes in the late Ordovician (Clarke et al. 2011), this basic mechanism has been adapted by evolution to face the challenges of photosynthesizing optimally in the diverse environmental niches found in the terrestrial biome.

Photosynthesis is a complex process that depends on the cooperative activity of many processes so it can be tuned or optimized along many dimensions, for example, resource use efficiencies such as for light, water, and mineral nutrients, responses to temperature, leaf architecture, and longevity, tolerance to abiotic stress, recovery from stress, etc. These axes for adaptation are individually complex; for example, light can vary in myriad ways (e.g. intensity, spectrum, periodicity, fluctuations over differing time scales) during the life of a leaf or plant. This has resulted in the evolution of variation in photosynthetic traits (e.g. Björkman and Holmgren 1963; Flood et al. 2011; Yamori et al. 2014; Faralli and Lawson 2020). Taking the maximum rate of photosynthetic CO_2_ fixation per unit leaf area (*P*_max,_ units *μ*mol m^−2^ s^−1^) as an example, for a plant using the C_3_ photosynthesis mechanism it has been estimated that *P*_max_ should be about 55 *μ*mol m^−2^ s^−1^ at an irradiance of 2,000 *μ*mol m^−2^ s^−1^ (Nobel 1991). Some C_3_ plants (e.g. desert winter annuals) achieve a slightly higher *P*_max_ [60 to 65 *μ*mol m^−2^ s^−1^ for *Chylismia* (formerly *Camissonia*) *claviformis* (Mooney et al. 1976) and *Palifoxia linearis* (Werk et al. 1983)]. In contrast, a typical C_3_ crop plant will have a *P*_max_ of 20 to 30 *μ*mol m^−2^ s^−1^ (Nobel 1991), deciduous forest tree species a *P*_max_ of about 10 *μ*mol m^−2^ s^−1^, while for permanently shaded rainforest understory species, *P*_max_ is 1 to 5 *μ*mol m^−2^ s^−1^.

Despite the variation for photosynthesis observed in natural systems, it has been argued that photosynthesis in crop plants cannot be improved because it has already been optimized by natural selection and evolution. This argument is, however, only partly correct because plants in nature do not experience the same pressures as plants in agriculture. The wide diversity of photosynthetic properties found in nature, including among the wild ancestors of crop plants, in broad terms is expected to represent different optima for photosynthesis adapted to different naturally occurring niches. These niches are, however, often limiting in various ways, for example, in water and nutrients (such as nitrogen and phosphate), and plants in nature usually experience strong competition from their neighbors (Theeuwen et al. 2022). In agriculture, however, competition with noncrop plant species is usually eliminated (or largely so), nutrients and water are often supplied, and attack from pests and diseases are managed, so far as possible, especially in intensive agriculture and protected horticulture. These agricultural environments do not have any perfectly natural analogs, so evolution will not have optimized photosynthesis for agriculture, and it can therefore still be improved in crop plants. In principle, therefore, photosynthesis is no different to those other plant properties that have been improved by domestication and breeding, despite their having been previously optimized by evolution in the wild ancestors of our crop plants. In the past, however, breeding for improved photosynthesis has occurred only to a limited extent, owing to the difficulty of phenotyping for photosynthesis alongside the complexity of the process (Theeuwen et al. 2022).

Variation in photosynthetic properties of land plants is a valuable resource in terms of understanding the operation and limitations of photosynthesis and finding photosynthetic syndromes or traits that could potentially be used to improve crop plant photosynthesis. The contribution that natural variation can make to understanding and improving photosynthesis can be divided into the following five broad categories:

1. The limits to the adaptability of plant photosynthesis to extreme habitats and environments.
2. Trait variation within or across species that facilitates the physiological analysis of traits and the identification of their underlying mechanisms.
3. The co-occurrence of subtraits that reveal syndromes of evolutionary adaptation of photosynthesis.
4. Physiological models or templates that serve as options for improving photosynthesis.
5. Sources of genetic (or allelic) variation that can be used to breed (including by means of novel plant breeding techniques) for improved photosynthesis.

In addition to providing a way to extend our knowledge of the operation and regulation of photosynthesis and the limits reached by evolutionary refinement of the process, the occurrence of natural variation of photosynthesis implies there must be an underlying genetic underpinning. If we can unravel this genetic basis of variation for photosynthetic traits we can begin to systematically breed for improved photosynthetic traits. The importance of linking variation in photosynthesis (i.e. phenotypic variation) to genetics is paramount whether or not novel plant breeding techniques or conventional breeding is used to genetically improve a plant. The problem is that while the genes for the headline components of photosynthesis (e.g. the subunits of PSII or PSI) are well known, the genetic basis of variation in many key photosynthetic properties is poorly understood, even when the physiology of that variation is well known. For example, the maximum rate of photosynthesis is correlated with numerous changes in the protein, lipid, and cofactor composition of the leaf, alongside anatomical differences (e.g. Osmond et al. 1980; Schulze and Chapin 1987; van Bel and Gamalei 1992; Brodribb et al. 2007; Taylor et al. 2022). Higher rates of photosynthesis are, for example, correlated with greater activities of Cyt *b_6_f* complex or Rubisco, but this increase in activity is largely achieved by there being more of these components per unit area and not by there being super Cyt *b_6_f* or Rubisco (i.e. complexes with substantially higher specific activity; e.g. Osmond et al. 1980; Makino et al. 1997; Schöttler et al. 2015; Miller et al. 2017). The genetic basis for how an increased amount of these complexes per unit area of leaf is achieved is poorly understood despite phenotypic variation in *P*_max_ being widely studied and encountered. The same can be said of other photosynthetic traits (or subtraits). This does not mean, however, that no natural variation exists for genes coding for the headline components of photosynthesis—such variation does exist (e.g. Prins et al. 2016; Prinzenberg et al. 2020) and can potentially be exploited.

If there is variation for a trait within a species, or a pool of species, that can be hybridized to give rise to genetically segregating offspring, then it is possible to correlate phenotypic variation with genomic variation using a mapping population and by this means identify genomic regions—QTL—within which a gene or genes that are causal for phenotypic variation are located (e.g. Harbinson et al. 2012; Theeuwen et al. 2022). Mapping populations can be constructed in various ways (Theeuwen et al. 2022), but critically they need to contain variation for the trait under investigation. The individuals comprising the population (typically 100 and often many more, depending on the nature of the population) must also be genomically mapped using markers (commonly SNPs) that essentially serve to describe the variation of the genome. If there is both genomic and phenotypic variation, these can be correlated and regions of genomic variation that are associated with phenotypic variation identified. A critical requirement is that of phenotyping; photosynthesis is strongly affected by the environment and is, in any case, difficult to measure rapidly and on the large scale needed to adequately phenotype a large population. This can be achieved by the large-scale use of portable gas analyzers or by using robotic chlorophyll fluorescence-based imaging systems (e.g. Flood et al. 2016). Comparing marker-assisted breeding and genomic selection on the one hand with genetic modification and gene editing on the other hand, the latter requires the identification of genes whose variation results in phenotypic difference, while the former can be carried out knowing only the association between phenotypic variation and variation in genomic markers (e.g. single-nucleotide markers or SNPs)—an approach that is widely used in commercial plant breeding [see Theeuwen et al. (2022) for a summary of QTLs and their use].

Improving photosynthesis is still a largely unexplored strategy for enhancing crop productivity, offering a means to expand crop improvement possibilities while sustaining yields. Even considering only the land plants (the embryophytes), photosynthesis has a wide range of properties many of which vary not only from species to species, but within a species. In some cases, these properties and their variation are not well explored. This natural variation for photosynthesis is, nonetheless, a valuable resource for improving crop photosynthetic properties, providing us with physiological templates and genetic resources with which we can improve crop photosynthesis using either conventional or novel plant breeding techniques. To make best use of this resource, we need to better understand the limitations on photosynthesis in the field and improve the tools needed for identifying phenotypic variation for photosynthesis. We also need better tools for identifying the causal genes underlying photosynthetic variation and, finally, develop strategies for applying these discoveries in crop improvement programs.

## Modeling photosynthesis

### By Xin-Guang Zhu

Engineering canopy photosynthesis, instead of leaf photosynthesis, is required to improve crop yield potential. Canopy photosynthesis is an integral of photosynthetic CO_2_ uptake rates for all leaves in a canopy, including those at the top, which usually receive high irradiance, and those in the lower layers, which usually receive low irradiance (Zhu et al. 2012). Earlier wheat breeding programs suggest that cultivars with a higher light-saturated rate of leaf photosynthesis are usually associated with a lower leaf area index, which consequently negates the positive impact of enhancing leaf photosynthetic rate (Evans and Dunstone 1970). Therefore, identifying engineering targets that can increase canopy photosynthesis becomes crucial to achieving the desired goal of increasing photosynthesis for greater yields.

Canopy photosynthesis is controlled by both the microclimate parameters inside a canopy, such as CO_2_, light, temperature, and humidity, and the photosynthetic properties of all leaves in a canopy. Although canopy photosynthesis can be measured with canopy chambers (Song et al. 2016), the development of accurate canopy photosynthesis models is needed for the precise dissection of the main players and regulatory factors. Various models have been developed, often based on 3D structural modeling and energy balance approaches and which differ in their levels of detail in canopy architecture and microclimate heterogeneity (dePury and Farquhar 1997; Song et al. 2017; Liu et al. 2021). These models have been used to identify key architectural parameters, such as optimal leaf area index and ideal leaf angle to enhance canopy photosynthesis (Song et al. 2013; Liu et al. 2021).

Besides microclimate, another factor controlling canopy photosynthesis is leaf photosynthetic rate, which shows large variation among species and between cultivars of the same species (van Bezouw et al. 2019). This variation is mainly attributed to differences in photosynthetic properties, e.g. Rubisco content, Rubisco activation state, CBB cycle enzyme activities, the abundance of electron transfer chain components, mesophyll and stomatal conductance (Long et al. 2015), and leaf anatomical features (Giuliani et al. 2013). To enable the precise dissection of factors controlling leaf photosynthetic rate, 3D reaction–diffusion models of leaf photosynthesis have been developed that effectively couple leaf anatomical features, CO_2_ diffusion processes, and light distribution inside a leaf (Xiao et al. 2016, 2022; Xiao and Zhu 2017). These models indicate that leaf biophysical and biochemical properties associated with photosynthesis play a dominant role in determining photosynthetic rates, while leaf anatomy makes a relatively minor contribution (Xiao et al. 2022).

**Figure 10.**
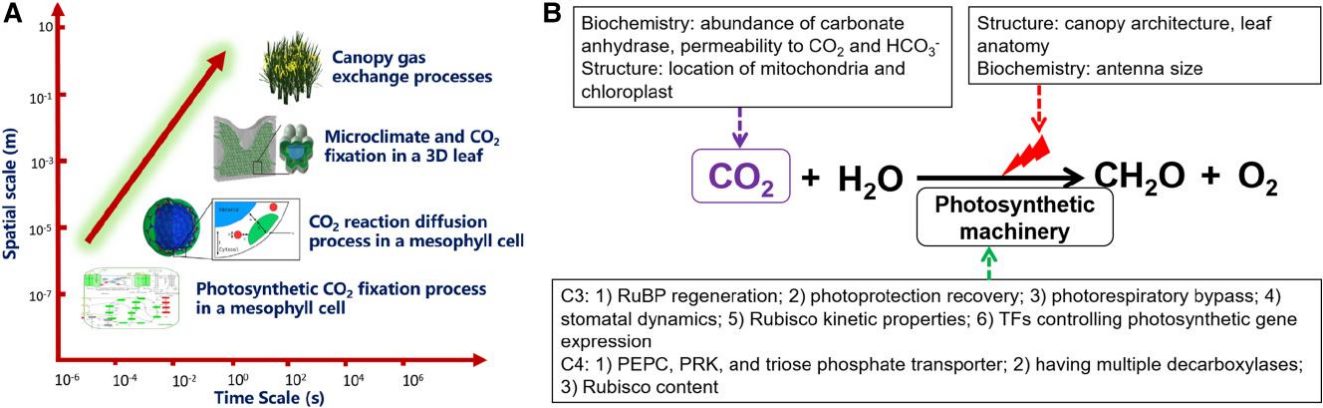
Systems approach to identify options to engineer photosynthesis for higher efficiency. **A)** Multi-scale models of photosynthesis. Models for photosynthesis at different organismal scales spanning from organelle, cell, leaf, up to canopy scales, have been developed. These models are used to define the architectural, anatomical, biophysical, and biochemical parameters controlling photosynthetic efficiency. **B)** Options to engineering photosynthesis for greater efficiency, divided into three categories: (i) increase the delivery of CO_2_, (ii) optimize light distribution across a canopy, and (iii) manipulate photosynthetic machinery.

There is a long history of modeling photosynthetic systems to identify critical proteins and enzymes and their biochemical and biophysical properties that control photosynthetic efficiency. Several modeling approaches have been used, e.g. systems of ordinary differential equations to model photosynthesis-related metabolic processes (Zhu et al. 2007, 2013), reaction–diffusion models to simulate the gas diffusion and the coupled reaction processes in a MC or leaf (Tholen and Zhu 2011; Tholen et al. 2012; Xiao and Zhu 2017), and ray-tracing algorithms to simulate light distribution inside a leaf (Xiao et al. 2016) (Fig. 10). Systems models of photosynthesis for C_3_, C_4_, and CAM leaves have been developed (Zhu et al. 2013; Wang et al. 2014b, 2023), as well as models simulating stomatal conductance (Buckley et al. 2003) and NPQ dynamics (Zaks et al. 2012).

Analyses with these systems models have generated tremendous insight into strategies for engineering photosynthesis. For example, a dynamic system model of canopy photosynthesis showed that faster recovery from photoprotective states improves canopy photosynthesis (Zhu et al. 2004), which in turn results in increased biomass production in both soybean and tobacco (Kromdijk et al. 2016; De Souza et al. 2022). Systems models of photorespiratory bypass suggest that decreased expression of PLGG1, a glycolate/glycerate transporter (Pick et al. 2013), results in an increased benefit of photorespiratory bypass and further increased photosynthetic CO_2_ uptake rate (Xin et al. 2015), which was later experimentally confirmed in tobacco (South et al. 2019) and rice (Shen et al. 2019a). Analysis with a systems model of C_3_ photosynthesis suggested that simultaneous overexpression of SBPase and FBPase results in increased photosynthesis, which was again later demonstrated in wheat (Zhu et al. 2007; Simkin et al. 2017a). Modeling together with field experiments has established a suite of engineering options that can be used to overcome limitations on photosynthesis. These options are discussed individually in the earlier sections. Here, we summarize them from the angle of access to substrates of photosynthesis, i.e. CO_2_ and light, and limitations due to the inefficiency of proteins or enzymes involved in photosynthesis (Fig. 10B).

Although there are successes of current models in guiding photosynthesis engineering, the suggested strategies do not always deliver the predicted increase in photosynthesis and biomass, as in the case of increasing the NPQ relaxation in Arabidopsis (Garcia-Molina and Leister 2020) and potato (Lehretz et al. 2022), or increasing the expression of SBPase in rice (Feng et al. 2007). One potential reason is that factors limiting photosynthesis might shift to different processes under different environments or in different species. For example, under high light, Rubisco has higher control over photosynthetic CO_2_ uptake, while under low light, components of the electron transfer chain exert higher control (Raines 2022); as a result, engineering a particular enzyme may not necessarily increase photosynthesis for a particular crop under a particular condition. However, this does not nullify the possibility that a particular step limits photosynthetic rates under other conditions, which therefore calls for field studies to test whether a particular engineering option will work for a particular plant or a particular cultivar. In some sense, once the original concept that a particular protein is limiting photosynthesis in model plant species is established, private enterprises might be better positioned to take the lead in systematically testing its application in different crops under different conditions through large-scale field testing.

It is worth pointing out that while the use of the systems model of photosynthesis has shown promise in guiding photosynthesis engineering for higher efficiency, this area of study is in its infancy. Great opportunities lay ahead to develop more advanced models to guide future engineering and design for higher photosynthetic efficiency. First, current models need to include a description of the acclimation of photosynthesis (e.g. expression, assembly, and degradation of proteins) to various environmental factors such as light, humidity, and temperature as well as internal factors like sink capacities. For example, a recent study shows that the ATP required for PSII repair processes is on average 4.6% of that used for photosynthetic carbon assimilation (Yi et al. 2022). Moreover, studies show that increasing the synthesis of the D1 protein, which has a high turnover rate (Li et al. 2018), enhances high photosynthetic rates (Chen et al. 2020). Second, the complete redesign of photosynthetic CO_2_ fixation and carbon metabolism has the potential to dramatically improve photosynthetic efficiency (Bar-Even et al. 2010; Trudeau et al. 2018). Third, systems modeling of excitation energy transfer and electron transfer processes need to incorporate the spatial organization of photosynthetic proteins and pigment–protein complexes in the thylakoid membrane. This information is crucial not only for understanding the principles underlying the high photosynthetic efficiency but also for identifying targets to optimize plants for even higher efficiency. Finally, the enzymes or proteins predicted as limiting factors by systems modeling can be directly correlated with sequence variations and molecular dynamics simulations to identify optimal genomic editing strategies for photosynthesis-related genes. With the rapid increase in computational power and the expanding capacity of photosynthesis models, we are entering an era of rational design not only for designing new pathways but also new proteins to significantly enhance the efficiency of crops.

## Smart canopy for enhanced crop yield and NUE

### By Young B. Cho and Donald R. Ort

In the dense monoculture system of current row crop agriculture, low-light use efficiency at the top of the crop canopy and limited light availability within the canopy conspire to limit carbon gain. The notion of a “smart canopy” concept was proposed for optimizing canopy photosynthesis (Ort et al. 2015). Proposed strategies for improving photosynthesis at the canopy level could benefit from considering a smart canopy concept in the context of optimization of nitrogen (N) distribution within the canopy and to provide promising target genes and enabling technologies.

Studies in optimization theory suggested that maximizing canopy photosynthesis requires N distribution proportional to the light availability within the canopy (Field 1983; Anten et al. 1995). Leaf N decreases gradually from the top to the bottom of the canopy, which is a crucial adaptation to declining light availability within the canopy (Hirose 2005; Pons 2016). The vertical distributions of light and N within canopies are described using the extinction coefficients for light (*K*_L_) and N (*K*_N_) (Hirose and Werger 1987). Canopy photosynthesis can be maximized by the optimal gradient of N and light (i.e. *K*_N_/*K*_L_ = 1) within canopies (Anten et al. 1995). A meta-analysis of canopy N distribution has revealed that the *K*_N_/*K*_L_ for most plant species is approximately 0.5 (Hikosaka et al. 2016), implying that improving canopy photosynthesis can be achieved by either reducing *K*_L_ (more uniform light distribution) or increasing *K*_N_ (less uniform N distribution) [see Niinemets (2023) for more details]. More than half of the leaf N is invested in the photosynthetic apparatus (Evans 1989), suggesting that “smart” regulation of photosynthetic apparatus within a canopy will mediate optimizing N distribution.

Various strategies have been proposed to enhance leaf and canopy photosynthesis, with many of them having been tested in field experiments. Evans and Clarke (2019) assessed the N costs of these strategies. For example, engineering Rubisco and ATP synthase was expected to have a high N cost, as they account for a significant portion of the N budget of the leaf (20% and 8%, respectively). On the other hand, overexpressing enzymes such as Cyt *b_6_f*, Psbs-VDE-ZEP, SBPase, and FBP aldolase incurred a medium N cost (Evans and Clarke 2019). The concept of a “smart canopy” can minimize the N cost by overexpressing rate-limiting enzymes only at the top of the canopy where sufficient light is available while repressing N investments in the enzymes where light is limited. This smart canopy strategy aims to maximize canopy-level photosynthesis while optimizing N distribution within the canopy. Interestingly, Evans and Clarke (2019) pointed out that one of the strategies for improving photosynthesis can save N, rather than incur additional N costs: reducing the light-capturing machinery such as chlorophyll and antenna proteins. Ort et al. (2015) proposed the smart canopy has more RCs and fewer antenna proteins at the top of the canopy, with the reverse arrangement in the lower canopy.

For agricultural purposes, crop plants overinvest in light capture while underinvesting in light utilization to optimize canopy carbon gain. Studies on low-chlorophyll crops have shown that many plants invest excessively in the production of chlorophyll and its associated LHC (Li et al. 2013; Gu et al. 2017b; Kirst et al. 2017; Slattery et al. 2017; Sakowska et al. 2018; Cho et al. 2023). One significant evolutionary advantage of overinvesting N in light capture is that it confers a selective advantage by shading potential competitors (Zhang et al. 1999). Even when a leaf is light-saturated and cannot utilize additional light, intercepting more light prevents potential competitors from receiving and benefiting from it; plants even produce more leaves than necessary to capture light (Srinivasan et al. 2017). Another benefit may be that many crops evolved under conditions of limited N, and thus, it is adaptive to sequester N whenever it is available, storing it in proteins like Rubisco to conserve this typically scarce resource (Denison et al. 2003). However, this N investment strategy is suboptimal for densely planted agricultural monocultures (Loomis 1993; Denison et al. 2003), where the main goal is to maximize net primary productivity in the field. A decrease of light capture, rather than an increase, to achieve improved light distribution in the canopy would benefit NUE and perhaps overall canopy photosynthesis (Ort et al. 2011).

Theory suggests an increase in total canopy photosynthesis if N saved by reducing leaf chlorophyll content was optimally reallocated to photosynthetic capacity that matched increased light levels within the reduced chlorophyll canopy (Song et al. 2017; Zhou et al. 2023). Experimental verification is needed to assess the impacts of these modifications on leaf and canopy photosynthesis. Theoretically, a 50% reduction in leaf chlorophyll and LHC could result in 7% to 9% savings in leaf N without negatively affecting canopy photosynthesis (Walker et al. 2018). The saved N from the reduction of chlorophyll could be utilized for the overexpression of rate-limiting enzymes in the CBB cycle, such as Rubisco, FBP aldolase, and SBPase (Zhu et al. 2007). Proposed strategies include different N investment approaches at various canopy levels, allocating N from lower to upper canopy leaves. Beyond the predicted advantages of re-investing saved N in increased photosynthetic capacity, Cho et al. (2023) demonstrated that reducing canopy chlorophyll levels in tobacco by up to 50% did not compromise carbon assimilation but increased seed N concentration by 7%, indicating that saved N from reducing chlorophyll and LHCs may be redirected toward seed N.

Multigene constructs, overexpressing a suite of rate-limiting enzymes such as Rubisco, SBPase, and FBP aldolase at the top of the canopy while downregulating overinvested enzymes in the lower canopy, will better optimize N distribution and improve canopy photosynthesis. These constructs should be regulated by distinct promoters customized for specific canopy heights. Perhaps the ratio of red/far-red (R/FR) light serves as a reasonable inducer, especially as the R/FR ratio decreases with canopy height. With the advent of optogenetics, blue light- and FR light-inducible gene expression systems have been tested in microorganisms (Gligorovski et al. 2023; Liu et al. 2023). However, these systems have not yet been tested in plants. The red light-inducible promoter has been implemented in a plant system, but it was found to turn off gene expression under white light (Ochoa-Fernandez et al. 2020), rendering it unsuitable for the intended purpose. Therefore, a canopy height-specific gene expression system should be further investigated under actual canopy conditions, preferably in field experiments.

The redistribution of the investment of N from light capture to photosynthetic capacity is a legitimate opportunity to improve light use efficiency and canopy carbon gain for the dense canopies of current row crop agriculture. Candidate targets for N savings and reinvestment have been identified and highlighted in this brief overview. The next critical steps involve adapting gene regulation and the validation of these predictions in target crop species in field experiments and ultimately in multi-location field trials to assess impacts on crop yield.

## Outlook

In this perspective, we have discussed key targets and methodologies designed to enhance photosynthetic efficiency, highlighting their significant potential for crop yield improvement. The next critical step is the integration of these approaches. By combining strategies such as optimizing Rubisco efficiency, boosting electron transport, introducing novel pigments and refining canopy structures, and improving stomatal regulation and photosynthetic responses to environmental fluctuations, we can harness their collective potential. Importantly, this integration must take into account the complex interplay of synergistic and antagonistic effects among these modifications to maximize their benefits for agricultural productivity. The combination of these strategies should then aim not only at stacking improvements but also at ensuring real benefits in terms of plant resilience and productivity in diverse farming conditions. This means not just mixing different improvements but also understanding how they work together in field conditions—how they affect plant growth, sink–source relationships, and the response to environmental stresses, and when and how photosynthesis limits crop productivity. This requires extending the focus beyond enhancing photosynthetic efficiency, incorporating traits that increase resilience to abiotic and biotic stresses, improve water use efficiency and NUE, and decrease the yield gap under fluctuating environmental conditions. Importantly, improving primary production creates flexibility in crop plant design. The increased carbon could be used to increase yield or, for example, it could be allocated, without a decrease in yield, to increased root biomass to improve agricultural sustainability by improving nutrient or water capture or by increasing soil organic carbon. The goal is to create crops that are more productive but also better suited to the diverse environments they grow in, directly addressing the pressing demands of global food security.

## Dive Curated Terms

The following phenotypic, genotypic, and functional terms are of significance to the work described in this paper:

EPF1 Gramene: AT2G20875
EPF1 Araport: AT2G20875

PGK1 Gramene: AT3G12780
PGK1 Araport: AT3G12780

PETC Gramene: AT4G03280
PETC Araport: AT4G03280

RCA1 Gramene: Q9ZT00
RCA1 Araport: Q9ZT00

PsbS Gramene: AT1G44575
PsbS Araport: AT1G44575

VDE Gramene: AT1G08550
VDE Araport: AT1G08550

ZEP Gramene: AT5G67030
ZEP Araport: AT5G67030

PsbS1 Gramene: LOC_Os01g64960
PsbS1 Araport: LOC_Os01g64960
